# Functional traits and phylogeny explain snake distribution in the world's largest dry forest ecoregion, the Gran Chaco

**DOI:** 10.1002/ece3.9503

**Published:** 2022-11-15

**Authors:** Hugo Cabral, Thaís B. Guedes, Diego J. Santana

**Affiliations:** ^1^ Programa de Pós‐Graduação em Biologia Animal Universidade Estadual Paulista São José do Rio Preto Brazil; ^2^ Instituto de Investigación Biológica del Paraguay Asunción Paraguay; ^3^ Mapinguari – Laboratório de Biogeografia e Sistemática de Anfíbios e Répteis, Instituto de Biociências Universidade Federal de Mato Grosso do Sul Campo Grande Brazil; ^4^ Departamento de Biologia Animal, Instituto de Biologia Universidade Estadual de Campinas Campinas Brazil; ^5^ Gothenburg Global Biodiversity Center and Department of Biological and Environmental Sciences University of Gothenburg Göteborg Sweden

**Keywords:** climate, ecology, habitat heterogeneity, macroecology, morphology, phylogenetic region, soil conditions, species traits

## Abstract

Macroecological studies describe large‐scale diversity patterns through analyses of species distribution patterns and allows us to elucidate how species differing in ecology, physical requirements, and life histories are distributed in a multidimensional space. These patterns of distributions can be explained by vegetation, and climatic factors, and are determined by historical and current factors. The continuous accumulation of information on the distribution patterns of species is essential to understand the history and evolution of the biota. In this study, we aimed to identify functional and evolutionary drivers that explain the geographic patterns of vertical stratification. We compiled morphological, ecological, and distribution data of 140 species of Chacoan snakes and constructed null models to map their geographic pattern. We used a range of environmental variables to assess which drivers are influencing these biogeographic patterns. Lastly, we used evolutionary data to build the first map of the phylogenetic regions of Chacoan snakes. We found a latitudinal pattern, with a marked verticality in the snake assemblies in the Chaco. Verticality and long‐tailed species richness increased in areas with high stratified habitats and stable temperature. Fossoriality is driven mainly by soil conditions, especially soils with fewer sand particles and less stratified habitat. Phylogenetic regions in the Chaco showed a marked latitudinal pattern, like that observed in the geographic pattern of verticality. The distribution pattern of Chacoan snakes also reflects their evolutionary history, with a marked phylogenetic regionalization.

## Graphical abstract

Chacoan snakes show a latitudinal pattern of distribution, with a marked verticality. Long‐tailed species richness and verticality increases in areas with high stratified habitats and stable temperature; and short‐tailed species richness and fossoriality is driven mainly by soil conditions, especially soils with fewer sand particles and less stratified habitat. The distribution pattern of Chacoan snakes also reflects their evolutionary history, with a marked phylogenetic regionalizaton.

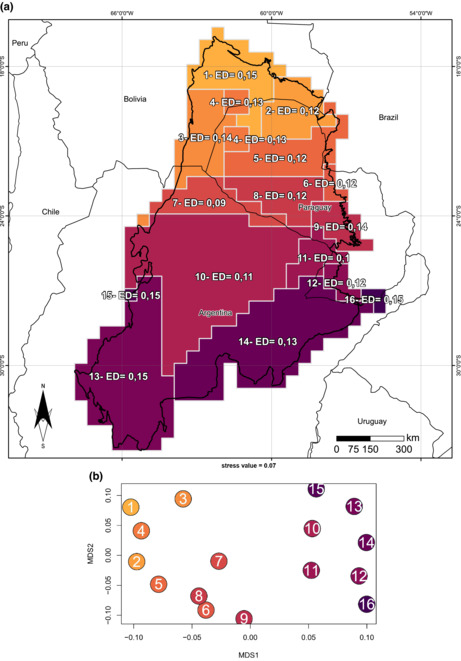

## 1 INTRODUCTION

Macroecological studies allow us to elucidate how species differing in ecology, physical requirements, and life histories are distributed in a multidimensional space (Gouveia, Hortal, et al., 2014; McGill et al., 2006). To access the interaction between species assemblages and better describe the structure of species geographic ranges, macroecological studies usually consider landscapes features (e.g., environmental and climate variables), intra‐ and interspecific variation in functional traits (e.g., morphological measurements and ecological habits), and the interaction between species assemblages to describe the structure of species geographic ranges (Gaston et al., 2008). These geographic ranges of species distribution are determined by historical and current factors, and changes in these patterns over time might result from environmental filtering, geographic distance, or natural barriers, and is reflected in the phylogenetic affinities of species (Daru et al., 2017; Hortal et al., 2012).

Historical and current factors like forest structure variables along with climatic factors can help us to understand the differences in species richness and diversity spatial patterns in several groups of organisms (Gatti et al., 2017; Roll et al., 2015). For example, vertical stratification (i.e., the gradient formed by the vertical structuring of the habitat) can be used in different ways by different groups of species (Pereira et al., 2010; Walther, 2002). It provides opportunities for individuals of arboreal species to exploit a greater number of niches (i.e., verticality) compared to any strictly ground‐dwelling species; these opportunities may result in morphological adaptations (Gaston et al., 2008; Harrington et al., 2018; Scheffers et al., 2014). Additionally, soil conditions associated with changes in climatic factors also explain species richness and it is another important factor that helps predict the diversity patterns in specific areas, as they are directly linked to microhabitat (Feldman & Meiri, 2014; Jackson et al., 2008; Moura et al., 2016; Scheffers et al., 2013, 2017). For instance, the body size of fossorial species decreases in areas with hottest temperature and low rainfall, making burrowing a critical ecological adaptation for survival in hot/dry areas (Burbrink & Myers, 2015; Feldman & Meiri, 2014).

Thus, functional traits along with environmental variables could also explain the variation in species within communities across space, species adaptation to different environments, and the trait–environment relationships to understand the processes that shape ecological communities (McGill et al., 2006; Violle et al., 2014; Weiher et al., 2011). For example, in tropical forested areas with medium temperature the diversity and richness of arboreal species is higher when comparing with hottest areas (Oliveira & Scheffers, 2019; Scheffers et al., 2013). This variation is followed by morphological adaptations, in which arboreal snakes show laterally compressed and slender bodies, long tails, and evident eyes (Guyer & Donnelly, 1990; Pizzatto et al., 2007); in contrast, fossorial snakes, are known by having smaller bodies, a relatively small tail, eyes not evident, and modification in body scales for burrowing (Cyriac & Kodandaramaiah, 2018; Kinlaw, 1999; Navas et al., 2004).

The knowledge of diversity patterns and its relationship with evolutionary processes in large South American open areas are scarce, which is until now mainly focused on tropical forests (Brusquetti et al., 2018; Werneck, 2011). This is the case of the Gran Chaco (hereafter Chaco), that is neglected in biodiversity research and harbors only few studies focusing mainly on conservation (Nori et al., 2016; Semper‐Pascual et al., 2018). It is worth mentioning that in recent decades, the Chaco has been suffering high deforestation rates (Hansen et al., 2013) and landscape degradation, making it a priority area for conservation (de la Sancha et al., 2021; Frate et al., 2015; Kuemmerle et al., 2017). Therefore, understanding the ecological and evolutionary processes that have shaped the diversity pattern of species distribution in this region becomes increasingly relevant.

In this paper, we assess what drives geographic patterns of vertical stratification of snakes in the Chaco. To address this, we (i) describe the geographic patterns of vertical stratification of snake species; (ii) determine what and how environmental drivers explain these patterns; and (iii) assess whether these patterns can also be recovered by the phylogenetic relationships of snake species distributed in the Chaco. We tested the three following predictions: (1) As environments become more vertically stratified, arboreality becomes a beneficial attribute allowed by morphological specializations for perching, nesting, and foraging on branches of trees (Gomes et al., 2009; Hildebrand & Goslow, 2001); we expected more arboreality and species richness in areas where habitats become more vertically stratified. (2) Temperature acts as a selective force on the body size of ecologically different snake species, resulting in miniaturization and morphological adaptations for burrowing in warmer areas (Olori & Bell, 2012), we expected that richness of fossorial species would be higher in warmer and drier areas with lower proportion of sand particles. (3) We also expect the geographic distribution pattern to be reflected in the phylogenetic relations of Chacoan snake species. This geographic pattern might be favored by the climatic and environmental characteristics of the Chaco, which would act historically as a filter for species dispersion (Daru et al., 2017).

## 2 MATERIAL AND METHODS

### 2.1 Study area

**FIGURE 1:**
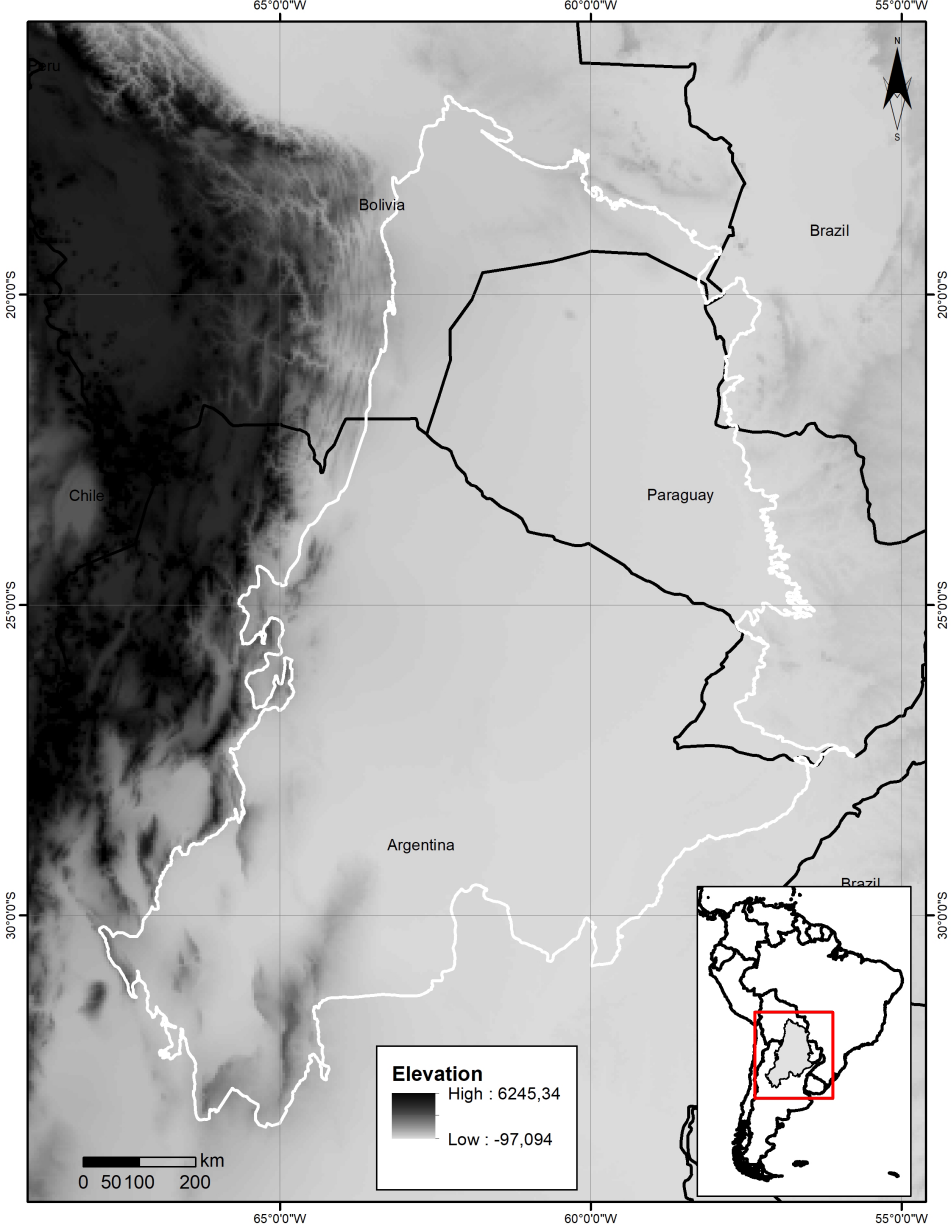
General view of the Gran Chaco. Geographical location of the Gran Chaco in South America (white polygon), with the elevation range (in meters) within the area. Notice the flatness topographic with the scarcity of elevation areas.

The Chaco is the largest continuous tropical dry forest in the world (Figure 1; Grau et al., 2008), formed from the Andean uplift, marine introgressions, and several alluvial systems (Gregory‐Wodzicki, 2000; Hernández et al., 2005). The most important characteristics of the Chaco are its climatic seasonality and geomorphology; the latter consists of an extensive sedimentary alluvial plain with soils derived from the accumulation of Quaternary sediments (Pennington et al., 2000; Prado, 1993). The Chaco is composed of xerophytic vegetation formed by a mosaic of grasslands, savannas, open woodlands, and thorn forests (Prado, 1993; Willig et al., 2000). In general, the Chaco shows little variation in elevation; the only topographic relief (1000–1200 m a.s.l.) occurs at the western border, between Argentina and Bolivia (Prado, 1993; Figure 1). Consequently, there appear to be no geographic barriers (e.g., large rivers or mountain ranges) that hinder the dispersal of organisms across the Chaco (Bucher, 1982).

### 2.2 Data source and preparation

We first downloaded distribution data of snakes with confirmed occurrence in the Chaco from the Global Assessment of Reptile Distributions (GARD; http://www.gardinitiative.org/data.html). We then reviewed the literature and assembled a database containing ecological and morphological data for all species. Ecological data included information on habit (i.e., aquatic, arboreal, semiarboreal, fossorial, semifossorial, and terrestrial). Morphological data included information on total length (TT), tail length (TL), snout‐vent length (SVL), TL to TT ratio, body mass, and eye diameter (Table S1). Arboreal snake species tend to have longer tails, with slender bodies, and bigger eyes associated with verticality specialization (Alencar et al., 2017; Harrington et al., 2018; Vitt & Caldwell, 2009); however, fossorial snake species tend to have smaller bodies, relatively small tail and reduction of eyes, modification of body scale structures (e.g., modified rostral scale in *Xenodon*, *Phimophis*, *Rena*; Cyriac & Kodandaramaiah, 2018; Kinlaw, 1999; Navas et al., 2004; Vitt & Caldwell, 2009).

We recorded 140 snake species from eight families (Boidae, Colubridae, Elapidae, Anomalepididae, Typhlopidae, Leptotyphlopidae, Aniliidae, and Viperidae) in the Chaco region. These species were distributed along 458 grid cells covering the entire Chaco. We provide the first and most extensive open dataset on ecological and morphological traits of all snake species recorded in the Chaco (Table S1). Of the 140 snake species from the Chaco, 70 are terrestrial, 22 fossorial, 21 semifossorial, 17 arboreal, and 10 semiarboreal. The total length of the Chacoan snakes ranged from 103 to 4000 mm, and the tail length ranged from 2 to 900 mm. In some cases, the tail represented almost 50% of the snakes' total length.

We used a range of environmental variables that potentially could reflect mechanisms that selects for species vertical niche: 19 climatic variables from WorldClim (at 2.5 min spatial resolution; Fick & Hijmans, 2017), three habitat productivity variables (Abatzoglou et al., 2018; Tucker et al., 2005), three habitat heterogeneity variables (Hansen et al., 2013; Tuanmu & Jetz, 2015), two soil variables (Hengl et al., 2017), and one topographic variable (Graham et al., 2014; for details see Table S2). All environmental variables were tested for multicollinearity using the variance inflation factor (VIF; Table S3), we also perform a Pearson correlation test, and no correlation was found between the variables cor >0.8 (Table S4). We excluded all variables with VIF values >10 (which indicate strong multicollinearity) keeping only those with VIFs were <10 (Zuur et al., 2010). After multicollinearity testing, the final set of variables included annual mean temperature, precipitation seasonality, annual actual evapotranspiration (AET), net primary productivity (NPP), normalized difference vegetation index (NDVI), evenness and homogeneity of the Enhanced Vegetation Index (EVI), tree cover (TC), volumetric percentage of coarse fragments (FRAG), and proportion of sand particles in the soil (SAND).

We created a presence–absence matrix using the snake distribution data and a 55 × 55 km grid cell superimposed on the Chaco region. As we were interested in the Chaco region, we chose a grid size that reflected macroecological patterns (Hurlbert & Jetz, 2007). We discarded species‐poor grid cells (those with less than five species), as they could affect the performance of the analyses (Moura et al., 2018; Oliveira & Scheffers, 2019).

### 2.3 Analyses

#### 2.3.1 Patterns of vertical stratification in snakes

To generate metrics of vertical stratification for each snake species, we performed a principal component analysis (PCA) using species‐specific body mass, eye diameter, verticality, and tail proportion (TL/TT; Figure S1). We then extracted the score that each species received on the first PCA axis (species‐specific mass, eye diameter, verticality, and tail proportion), which best explained our data. We used these scores as a metric; positive values indicated greater arboreality, and negative values indicated greater fossoriality. We used three verticality metrics as response variables: vertical niche or habitat (hereafter verticality), tail proportion, and PCA scores (fossoriality metrics) since it reflects the level of specialization of species, and therefore to their functional traits (Alencar et al., 2017; Harrington et al., 2018; Kinlaw, 1999; Oliveira & Scheffers, 2019; Webb et al., 2000), also the pattern of fossoriality is reflected in the first axis of the PCA, without overlapping and gradually.

To assess the geographical richness pattern of verticality, tail proportion, and fossoriality, we first constructed null models to control the effect of species richness on verticality metrics and maintain internal structure of species richness while randomizing the metrics (Swenson, 2014). We adopted a weighted sampling scheme to control the influence of widespread species, as they can overestimate the results compared to species with restricted distributions (Oliveira & Scheffers, 2019; Safi et al., 2013). We repeated this procedure 1000 times to generate a null distribution of verticality, tail proportion, and fossoriality and calculated the standardized effect size (SES) between the observed values and null values using the formula: SES = observed values – mean(null)/Standard Deviation(null). Hereafter, we refer to the SES values as verticality, tail proportion, or fossoriality. For verticality, positive values indicate assemblies containing more arboreal species, whereas negative values indicate assemblies containing more fossorial species. For tail proportion positive values indicate higher richness of snakes with longer tails and for the fossoriality metrics, positive values indicate higher richness of arboreality and negative values higher richness of fossoriality.

#### 2.3.2 Environmental drivers of vertical stratification

To assess the environmental drivers that explain the geographical richness pattern of verticality, tail proportion and fossoriality we fitted spatial autoregressive (SAR) models using all possible combinations of predictor variables and applied a model averaging approach (Burnham & Anderson, 2007; Moura et al., 2018). Spatial autoregressive models incorporate spatial autocorrelation with weighted matrices that specify the interaction strength between neighboring sites (Dormann et al., 2007). We tested our three metrics in a multimodel inference framework to investigate the contribution of our predictor variables. We constructed connectivity matrices for each metric (verticality, tail proportion, and fossoriality), defined by the distance at which Moran's *I* was strongest. Moran's *I* analyzes the spatial autocorrelation variations between neighboring sites, measuring whether there is a spatial autocorrelation between variables (Moran, 1950).

We assessed the effects of multiple weighting functions when defining spatial weight matrices and selected the one that best accommodated the spatial structure present in the variables (Oliveira & Scheffers, 2019). Residuals from the models were tested for significant autocorrelation using Moran's *I* (Figure S2), but no spatial autocorrelation was found in the spatial structures and metrics. We ranked the support of each model using Akaike's information criterion corrected (AICc) the weighted average model (wAICc) and used the standardized coefficient across all SAR models to compare with our explanatory variables (Burnham & Anderson, 2007). To estimate the variance explained by the averaged models, we calculated the pseudo‐*R* ^2^ as the squared Pearson correlation coefficient between the weighted fitted values and the observed values (Kissling & Carl, 2008). Higher values of pseudo‐*R* ^2^ indicate better model fit. Statistical analysis was performed in R environment version 4.0.4 (R Core Team, 2019) using *spdep* (Bivand, 2020), *adespatial* (Dray et al., 2020), and *MuMIn* (Bartón, 2020) packages.

Arboreal snakes are generally larger than nonarboreal snakes, and there is evidence that the body size of arboreal snakes is more constrained than in nonarboreal ones (Harrington et al., 2018). Thus, to test the robustness of our results, we repeated the analysis of environmental drivers after removing genera containing mainly arboreal species (*Chironius*, *Corallus*, *Dipsas*, *Imantodes*, *Leptodeira*, *Leptophis*, *Siphlophis*, and *Spillotes*). We adopted this approach because including these mainly arboreal genera could inflate the results in sites with higher arboreal diversity.

#### 2.3.3 Phylogenetic regionalization

To explore whether the patterns of vertical stratification of snakes (verticality and fossoriality) have an evolutionary component, we performed a phylogenetic regionalization analysis using phylogenetic relationships and distribution data of Chacoan snake species. We used the most comprehensive phylogeny of squamates to date (Tonini et al., 2016), which includes all snake species recorded in the Chaco. We pruned the phylogeny to precisely match all snake species recorded in the Chaco (Figure S8) using the package *picante* (Kembel et al., 2010) in the R programming language version 4.0.4 (R Core Team, 2019). Phylogenetic regionalization (phyloregion) procedures were performed using *phyloregion* package (Daru et al., 2020) in R environment version 4.0.4 (R Core Team, 2019). This package uses a community matrix in a sparse format with phylogenetic information. The ‘optimal phyloregion’ function allows the user to find the optimal number of phylogenetic regions, cluster, and plot the results. To perform the phyloregion analysis, we used the presence–absence matrix and the snake phylogeny (see section 2.2 in section 2).

## 3 RESULTS

### 3.1 Patterns of vertical stratification of snakes

**FIGURE 2:**
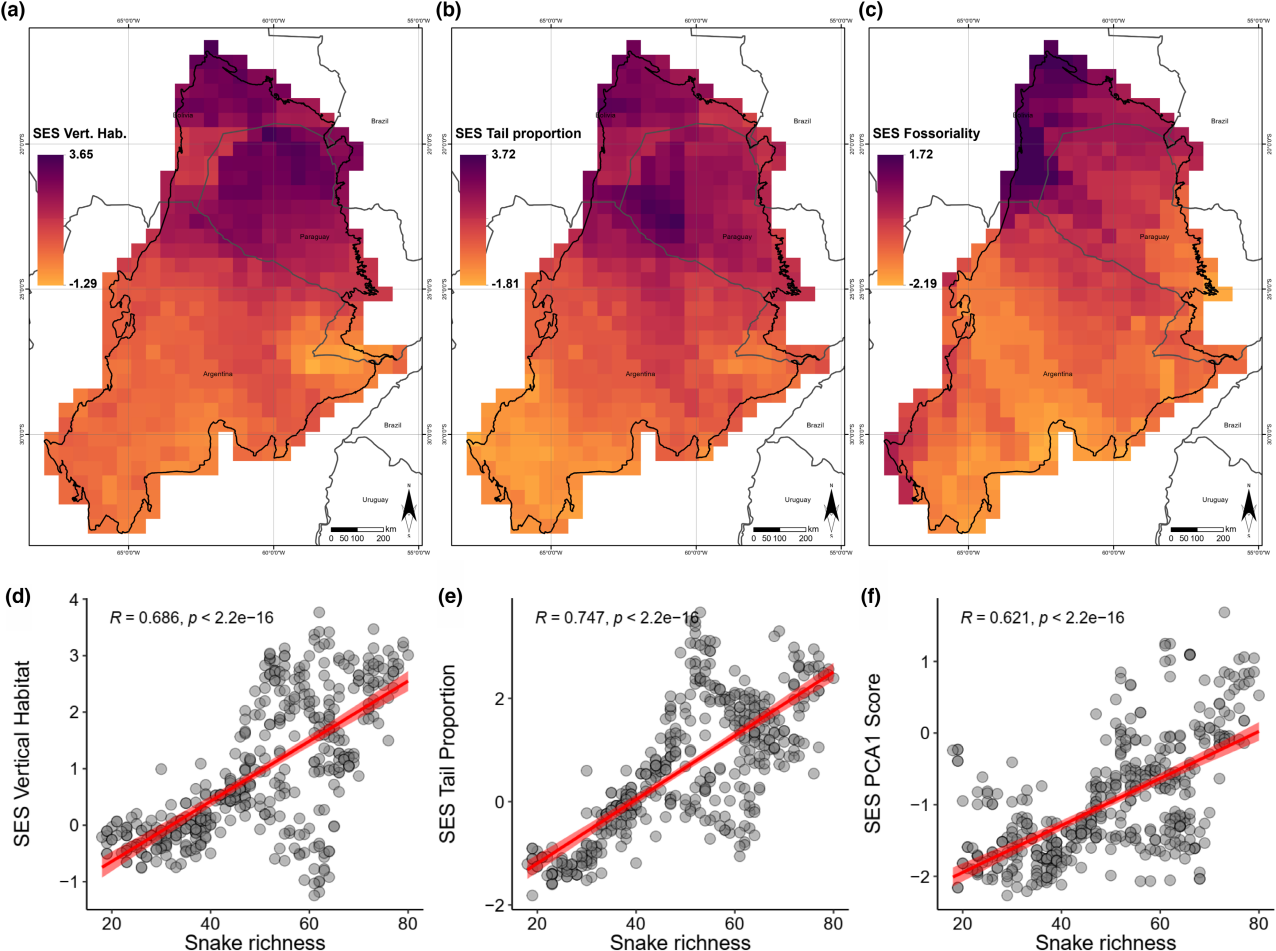
Geographical pattern and correlation of species richness with metrics used in the analysis: (a) geographical pattern of verticality; (b) geographical pattern of tail proportion; (c) geographical pattern of fossoriality; (d) correlation between species richness and verticality; (e) correlation with tail proportion; and (f) correlation with fossoriality.

We found a latitudinal pattern of vertical stratification of snakes, with arboreality increasing toward the north and fossoriality becoming more restricted in the south Chaco (Figure 2a–c). Additionally, a small patch southwest of the Chaco can be noticed with the presence of terrestrial and arboreal species (Figure 2c). We also found a marked pattern of verticality in the snake distribution of assemblages in the Chaco, increasing in the north and decreasing in the south. Moreover, we found a positive correlation between species richness and verticality, tail proportion, and fossoriality (Figure 2d–f).

### 3.2 Environmental drivers of vertical stratification

**FIGURE 3:**
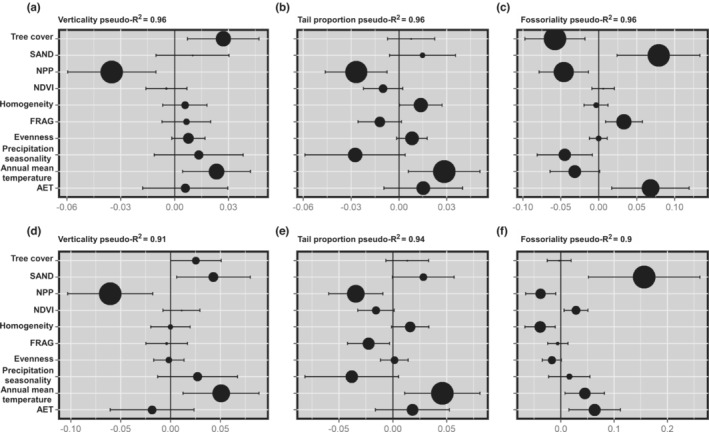
Model averaging showing the influence of each predictor variables explaining verticality habitat, tail proportion and fossoriality. Results derived from the SAR models, presenting all possible combinations of variables: (a‐c) the entire dataset results are presented; (e‐f) results from the sensitivity analysis. The size of circles represents the relative importance of each variable, plotted against the average standardized coefficients, and pseudo‐R^2^.

The averaged models explained more than 90% of the variation in verticality, tail proportion, and fossoriality, as indicated by the pseudo‐*R* ^2^ (Figure 3a–c). Verticality was negatively correlated with NPP (*r* = −.453, *p* < 2.2 e‐16), but positively correlated with TC (*r* = .619, *p* < 2.2 e‐16) and annual mean temperature (*r* = .794, *p* < 2.2 e‐16; Figure S3). The NPP, TC, and annual mean temperature showed the strongest effect on the model (Figure 3a), it was followed by homogeneity, evenness, and AET. Verticality and species richness increase in areas in northern Chaco, and decrease gradually to the south, indicating that areas with greater vegetation structure have more species that are more vertically stratified, when the habitat becomes less heterogeneous, richness of arboreal species and richness decrease (Figure 2a–c). For tail proportion, the variables with the strongest effect on the model were NPP, annual mean temperature, and precipitation seasonality (Figure 3b). Tail proportion was positively correlated with annual mean temperature (*r* = .816, *p* < 2.2e‐16) and homogeneity (*r* = .287, *p* < 5.02e‐10), but negatively correlated with NPP (*r* = −.24, *p* < 2.42e‐07), actual evapotranspiration (*r* = −.136, *p* < .00384), precipitation seasonality (*r* = −.0611, *p* < .194), evenness of vegetation (*p* = −.151, *p* < .00127), and FRAG (*r* = −.501, *p* < 2.2e‐16; Figure S3). Long‐tailed species are associated with habitats with high vertical stratification and stable climates, this is somehow related to arboreality since arboreal species tend to have longer tails (Figure 2b and Figure S3). The most important variables affecting fossoriality were SAND, TC, and NPP (Figure 3c). Fossoriality was positively correlated with SAND (*r* = .315, *p* < 5.03e‐12), TC (*r* = .487, *p* < 2.2e‐16), annual mean temperature (*p* = .658, *p* < 2.2e‐16), and precipitation seasonality (*r* = .107, *p* < .0229) but negatively correlated with NPP (*r* = −.264, *p* < 1.24 e‐08), FRAG (*r* = −.267, *p* < 6.85e‐09), and actual evapotranspiration (*r* = −.297, *r* < 1.16e‐10; Figure S3). Fossoriality was frequent in the southern part of the Chaco where the habitats is characterized by soils with proportionally fewer sand particles, with less stratified habitats (Figure 2c).

The analysis excluding the mostly arboreal genera (*Chironius*, *Corallus*, *Dipsas*, *Imantodes*, *Leptodeira*, *Leptophis*, *Siphlophis*, and *Spillotes*) showed the same general pattern, with slight differences (Figure 3d–f, Figures S4 and S5). For verticality, the variables with the strongest effect on the model were NPP and annual mean temperature (Figure 3d). For tail proportion, the variables with the strongest effect were NPP, annual mean temperature, and precipitation seasonality (Figure 3e). However, for fossoriality, the SAND was the most important explanatory variable (Figure 3f). This result was expected due to the biology of fossorial species, which are adapted to underground life. We also observed that tree cover had almost no effect on fossoriality, which complements the previous result (Figure 3f). Fossorial species spend most of the time buried and exhibit morphological adaptations for living underground such as relatively small bodies and short tails (Figure 2e, Table S1).

### 3.3 Phylogenetic regionalization

**FIGURE 4:**
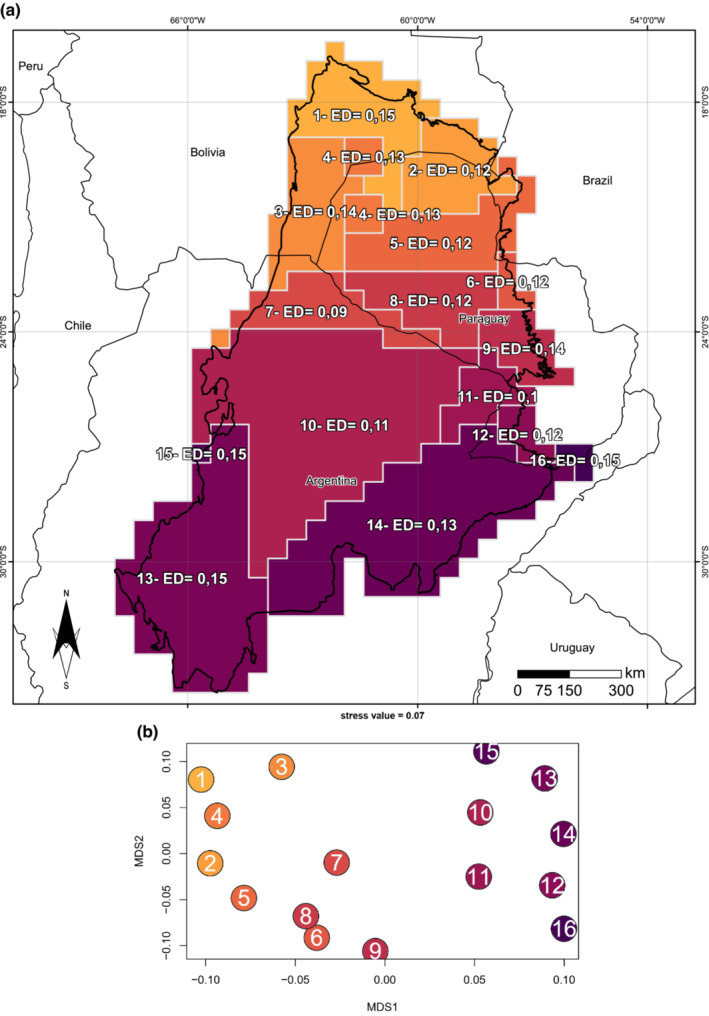
Phylogenetic regionalization of snakes of the Gran Chaco: (a) all 16 phylogenetic regions showing the evolutionary affinities with evolutionary distinctness (ED) values of snake’s assemblages; and (b) ordination of phylogenetic region in non‐metric multidimensional scaling (NMDS) space shows that different phylogenetic region differs in the evolutionary uniqueness, three groups can be noticed, following the ordination: phylogenetic region 1 to 5, 6 to 9 and 10 to 16.

We recovered 16 phylogenetic regions in the Chaco, with a marked north–south pattern (Figure 4a), similar to that observed for the geographic pattern of verticality (Figure 2). Lineages of arboreal species is higher in the northern part of the Chaco where habitat heterogeneity is higher and stratified. These arboreal lineages of species decrease as we move toward south, where lineage of fossoriality becomes more common, and the habitat heterogeneity decreases becoming less stratified. In the north of the Chaco there are more clades associated with Colubrinae than in the south, especially the arboreal genus *Chironius*. However, in the south, lineages/clades of fossorial species (e.g., Scolecophidea, Elapomophini) become more common. Phylogenetic regions to the north are evolutionarily distinct from those to the south Figure 4a. Clades of mostly terrestrial species remain similar across phylogenetic regions.

Phylogenetic region 1, 13, 15, 16 have the highest evolutionary distinctness ED = 0.15, follow by phylogenetic region 3 and 9 with ED = 0.14 (Figure 4a). These phylogenetic regions are in the north and south part of the Chaco, phylogenetic region with lowest ED is located mostly at the center of our study area (Figure 4a). From these 16 regions, three main groups were identified, showing that different regions differ strongly in evolutionary distinctiveness (Figure 4b, Figures S6 and S7). These regions also showed a similar relationship between the evolution and environmental influence on the Chacoan snakes, shaping their actual distribution pattern. The NMDS ordination show this same pattern, suggesting an important division within the snake fauna along the north–south axis of southern.

## 4 DISCUSSION

Our results recovered an evident geographic pattern of snake distribution related to verticality and fossoriality, in which the species richness of arboreal snakes is restricted to the northern Chaco where the habitat is vertically stratified and heterogeneity increases; while, in contrast, species richness of fossorial snakes is more common in the southern Chaco where habitat become less stratified and more heterogeneous (see Figure 2a–c,f). The northern Chaco is strongly influenced by forested areas with more vertically stratified habitats, such as the Amazon, Chiquitania Dry Forest, and Andean Forest (Spichiger et al., 1995). In contrast, the southern Chaco is more influenced by open areas, such as the Pampas and Patagonia (Nori et al., 2011). This pattern corroborates that previously recorded for amphibians, in which fossorial species are more frequent in deserts and xeric environments characterized by dry climate, with high temperatures and low precipitation (Bolochio et al., 2020; Oliveira & Scheffers, 2019).

As we predicted, verticality increases where areas are more vertically stratified with more habitat heterogeneity, and is driven by climatic factors and habitat conditions, especially tree cover and annual mean temperature (Figure 3a). Vegetation structure and forest stratifications allow the arrangement of more niches and resources, enabling the co‐occurrence of several arboreal species and increasing canopy richness (Gouveia, Villalobos, et al., 2014; Scheffers et al., 2013). Arboreal species tend to have longer tails, a morphological adaptation to arboreality (Harrington et al., 2018). Long tails are positively correlated with arboreality but negatively correlated with fossoriality, that is, fossorial species tend to have smaller bodies and shorter tails (Cyriac & Kodandaramaiah, 2018; Kinlaw, 1999; Navas et al., 2004; Wu et al., 2015). Like our results for snakes, vertically stratified habitats are known to play an important role in determining patterns of species richness and abundance in many other organisms worldwide (Gouveia, Hortal, et al., 2014; Stein et al., 2014). Climbing trees allows access to different microhabitats and resources, and this habit use is facilitated by morphological (functional) adaptations for survival and locomotion in snakes (Harrington et al., 2018; Scheffers et al., 2013, 2017).

In detail, our results also suggest that fossoriality in Chacoan snakes is mainly driven by soil conditions (Figure 3d–f), corroborating our second prediction. The number of fossorial snake species tends to increase in areas with lower proportions of sand particles and warmer areas (Figure 3f). In the Chaco, fossorial species tend to be smaller and have shorter tails than surface dwellers, possibly for burrowing and living underground, sheltering from extreme conditions, protecting, and feeding. This region, also known as the South American Pole Heat, has the highest absolute temperature in South America (maximum of 48.9°C) and scarce precipitation (Prado, 1993; Prohaska, 1959). These characteristics result in a marked dry and rainy season, a soil composed of fine sediments, and a rarity of rocky outcrops, which collectively drive and enable the species' ability to burrow (Kinlaw, 1999). Consequently, these habitat characteristics may select for fossoriality acting as a barrier to the dispersion of arboreal snakes. These observations in the Chaco are likely due to environmental filtering, affecting the distribution of species (Scheffers et al., 2017). Fossoriality has evolved multiple times in snakes (Cyriac & Kodandaramaiah, 2018), apparently associated with diversification related to habitat adaptation (Moen et al., 2016; Wiens et al., 2015). In the Chaco, fossoriality occurs in Colubridae (Colubrinae and Dipsadinae), Elapidae (Elapinae), and Scolecophidia (Anomalepididae, Leptotyphlopidae, and Typhlopiddae; Figure S8); the latter is the only snake group containing only fossorial species. However, the distribution of this specialized fossorial group is still poorly known. Thus, certain habitat conditions may favor the aggregation of fossorial taxa (Clinchy et al., 2002; O'Brien et al., 2008).

Finally, as predicted, we found that the spatial pattern reflects the phylogenetic affinities of the species. In the Chaco, the geographic pattern of verticality and fossoriality is also reflected in the phylogenetic regions we found (Figure 4). This result is due to specialization in a single habitat component, allowing fossorial species to efficiently exploit underused parts of the available resource base (Greenville & Dickman, 2009). Another explanation could be that the limited dispersal capacity of fossorial species makes them geographically restricted, resulting in evolutionary and geographically distant phyloregions (Daru et al., 2017). Furthermore, species distribution and range limits are determined by biotic and abiotic factors, movement, population dynamics, and intraspecific variability, which are related to the physiological tolerances of species (Gouveia, Hortal, et al., 2014). This should facilitate an increase in species richness of local communities, allowing specialist taxa to achieve high density in their selected habitats (Pianka, 1994). Moreover, environmental filtering may be influencing the actual species distribution, showing a heterogeneous phyloregion with marked regionalization (Daru et al., 2017).

Our study fills an important gap in the knowledge of the distribution of snake species in the Chaco and the macroecology of South American open areas. We identified phyloregions, which are important for a better understanding of what shapes the distribution of biodiversity, and we provide information on the ecological properties of the species that comprise them (Daru et al., 2017). Therefore, phyloregions may function as an important unit for the conservation of evolutionary history in overlooked areas such as the Chaco (Daru et al., 2017; Nori et al., 2016). This suggestion is especially relevant as Chacoan vertebrates are facing conservation issues, with a poor representation in protected areas (Andrade‐Díaz et al., 2019; Nori et al., 2016).

In conclusion, this is the first integrative approach study investigating macroecological and biogeographic patterns and the phylogenetic relationships of the Chacoan snakes, a unique area (Szumik et al., 2012). We found that as arboreality increases toward the north, fossoriality increases toward the south. The environmental pattern represents a marked vertical stratification in the Chaco, increasing as habitat heterogeneity increases. Fossoriality is strongly correlated with and thus driven by soil conditions. The distribution pattern in the Chaco is also explained by the evolutionary history of snakes in this region, with a marked phylogenetic regionalization.

## AUTHOR CONTRIBUTIONS

**Hugo Cabral:** Conceptualization (equal); data curation (equal); formal analysis (lead); funding acquisition (equal); investigation (equal); methodology (lead); project administration (equal); resources (equal); software (equal); validation (equal); visualization (equal); writing – original draft (lead); writing – review and editing (equal). **Thaís B. Guedes:** Conceptualization (equal); investigation (equal); methodology (equal); validation (equal); visualization (equal); writing – review and editing (equal). **Diego J. Santana:** Conceptualization (equal); funding acquisition (equal); investigation (equal); methodology (equal); project administration (equal); resources (equal); supervision (equal); validation (equal); visualization (equal); writing – review and editing (equal).

## CONFLICT OF INTEREST

The authors have declared that no competing interests exists.

## Supporting information


Appendix S1

## Data Availability

Data from reptiles are available at GARD. The code used here is available on https://github.com/hugodryas/Fossoriality_Chaco. Additional information supporting all results presented in this paper are available in the Supporting Information section.
