# Supplementary material for: Functional traits and phylogeny explain snake distribution in the world's largest dry forest ecoregion, the Gran Chaco
Source: Ecol Evol. 2022 Nov 15;12(11):e9503. doi: 10.1002/ece3.9503 (PMC9666913; doi:10.1002/ece3.9503)
Supplement: Supplementary file 1 — Appendix S1 [file ECE3-12-e9503-s001.docx]

**Supporting Information**

**Table S1.** Dataset of species and traits used in the analysis. In the case of snout-vent length (SVL), tail length (TL) and body length we used the maximum measure, prioritizing measures of one individual, when possible. For the size of the eyes, we compared the size of the supralabial just below the eye and his relations with the head, the eye size/diameter was classified in small = 1, medium = 2 and large = 3, body mass was log10 transform. Mass values were taken from (Feldman *et al.*, 2016). For habitat use, we reclassify the categories into a range of 1 to 3: fossorial = 1, semifossorial = 1.5, aquatic and terrestrial = 2, semiarboreal = 2.5, arboreal = 3 (see Oliveira & Scheffers, 2019). Tail proportion was calculated in relation to total length. Measures are in millimeters and mass in grams. A total of 184 studies were compiled, including papers, book chapters, books, thesis, and unpublish data. Unpublish data of species measurements were taken from the first author's personal database and are marked with an asterisk (*).

| Binomial | Habitat | BodyLength | SVL | TL | BodyMass | EyeSize | References |
| --- | --- | --- | --- | --- | --- | --- | --- |
| *Amerotyphlops brongersmianus* | Fossorial | 325 | 207 | 7 | 18.4 | Small | (Adalsteinsson et al., 2009; Avila et al., 2006; J. R. Dixon & Hendricks, 1979; J. R. Dixon & Kofron, 2009; Graboski et al., 2019; Hedges et al., 2014; Reis Martins et al., 2010; Vanzolini, 1968) |
| *Amerotyphlops reticulatus* | Fossorial | 522 | 496 | 22 | 105.8 | Small | (J. R. Dixon & Hendricks, 1979; J. R. Dixon & Kofron, 2009; Graboski et al., 2019; Hedges et al., 2014; Vanzolini, 1968) |
| *Anilius scytale* | Fossorial | 904 | 869 | 35 | 340.1 | Small | (Marcio Martins et al., 2008; Marcio Martins & Oliveira, 1998; Maschio et al., 2007) |
| *Apostolepis ambiniger* | Fossorial | 500 | 460 | 40 | 50.2 | Small | (de Lema, 2001; França et al., 2018; Nogueira et al., 2012) |
| *Apostolepis assimilis* | Fossorial | 200 | 186 | 14 | 29.1 | Small | (Cabral & Perez, 2015; Cei, 1993; Alejandro R. Giraudo & Scrocchi, 1998; M. Harvey, 1999) |
| *Apostolepis breviceps* | Fossorial | 419 | 387 | 32 | 9.3 | Small | (M. B. Harvey, 2008) |
| *Apostolepis dimidiata** | Fossorial | 571 | 518 | 53 | 90.6 | Small | (Cabral, De Lema, et al., 2017; Cei, 1993; Alejandro R. Giraudo & Scrocchi, 1998; M. Harvey, 1999; M. B. Harvey et al., 2008) |
| *Apostolepis dorbignyi* | Fossorial | 411 | 362 | 49 | 37.4 | Small | (M. Harvey, 1999; Loebmann & de Lema, 2012) |
| *Apostolepis intermedia* | Fossorial | 351 | 319 | 32 | 6.6 | Small | (Nelson Rufino De Albuquerque & De Lema, 2012; Entiauspe-Neto et al., 2014) |
| *Apostolepis multicincta* | Fossorial | 384 | 333 | 51 | 40.7 | Small | (Embert & Reichle, 2003; França et al., 2018; M. Harvey, 1999) |
| *Apostolepis nigroterminata* | Fossorial | 387 | 370 | 17 | 22.8 | Small | (F. M. Dos Santos et al., 2018; M. Harvey, 1999) |
| *Apostolepis vittata* | Fossorial | 493 | 460 | 33 | 39.8 | Small | (França et al., 2018; M. Harvey, 1999; Peters & Orejas-Miranda, 1972) |
| *Atractus bocki* | Fossorial | 447 | 370 | 77 | 18.4 | Small | (Cei, 1993; Paulo Passos et al., 2009) |
| *Atractus latifrons* | Fossorial | 613 | 521 | 92 | 52.5 | Small | (Almeida et al., 2014; Hoogmoed, 1980; M Martins & Oliveira, 1993) |
| *Atractus paraguayensis** | Fossorial | 502 | 490 | 12.7 | 37.9 | Small | (Cabral, 2014; A. R. Giraudo & Scrocchi, 2000; P Passos et al., 2010) |
| *Atractus reticulatus* | Fossorial | 346 | 295 | 51 | 20.7 | Small | (Balestrin & Di-Bernardo, 2005; A. R. Giraudo & Scrocchi, 2000; P Passos et al., 2010; Pizzatto, Jordão, et al., 2008) |
| *Boa constrictor* | Semiarboreal | 4000 | 3777 | 223 | 35283.8 | Large | (Bertona & Chiaraviglio, 2003; Boback, 2005, 2006) |
| *Boiruna maculata* | Terrestrial | 1640 | 1340 | 300 | 748.8 | Medium | (Cei, 1993; Pizzatto, 2005; Scott et al., 2006; H Zaher, 1996) |
| *Bothrops alternatus* | Terrestrial | 1216 | 1114 | 102 | 2399.7 | Medium | (Cabral, Rojas, et al., 2017; Cei, 1993) |
| *Bothrops ammodytoides* | Terrestrial | 712 | 570 | 80 | 521.2 | Medium | (Carrasco et al., 2010; Cei, 1993) |
| *Bothrops diporus* | Terrestrial | 1000 | 860 | 140 | 687.8 | Medium | (V. X. da Silva & Rodrigues, 2008; M. B. Harvey et al., 2005) |
| *Bothrops itapetiningae* | Terrestrial | 576 | 510 | 66 | 141.9 | Medium | (Leão et al., 2014) |
| *Bothrops jararacussu* | Terrestrial | 2000 | 1800 | 200 | 5169.5 | Medium | (Cei, 1993; A. Giraudo, 2002; M. B. Harvey et al., 2005) |
| *Bothrops matogrossensis* | Semiarboreal | 1001 | 878 | 123 | 1118.3 | Medium | (V. X. da Silva & Rodrigues, 2008; M. B. Harvey et al., 2005; Monteiro et al., 2006) |
| *Bothrops moojeni* | Terrestrial | 1060 | 954 | 106 | 5883.4 | Médium | (Cei, 1993; M. B. Harvey et al., 2005; Nogueira et al., 2003) |
| *Bothrops pauloensis* | Terrestrial | 1001 | 878 | 123 | 470 | Médium | (V. X. da Silva & Rodrigues, 2008; Valdujo et al., 2002) |
| *Chironius bicarinatus* | Arboreal | 1390 | 890 | 500 | 521.4 | Large | (Almeida-Santos & Marques, 2002; R. J. Bailey, 1955; Cacciali & Cabral, 2015; J. Dixon et al., 1993; O. A. V. Marques et al., 2009) |
| *Chironius exoletus* | Arboreal | 1531 | 973 | 558 | 353.7 | Large | (R. J. Bailey, 1955; Cacciali & Cabral, 2015; J. Dixon et al., 1993; Hamdan & Fernandes, 2015; Torres-Carvajal et al., 2019) |
| *Chironius flavolineatus* | Arboreal | 1404 | 880 | 524 | 186.2 | Large | (Cacciali & Cabral, 2015; J. Dixon et al., 1993; Hamdan & Fernandes, 2015; Hamdan et al., 2014) |
| *Chironius fuscus* | Arboreal | 1597 | 1095 | 502 | 384.8 | Large | (de Souza Filho et al., 2012; J. Dixon et al., 1993; Hollis, 2006; Torres-Carvajal et al., 2019) |
| *Chironius laurenti* | Arboreal | 2152 | 1445 | 707 | 820.5 | Large | (J. Dixon et al., 1993; Torres-Carvajal et al., 2019) |
| *Chironius maculoventris* | Semiarboreal | 1155 | 785 | 370 | 183.5 | Large | (Cacciali & Cabral, 2015; J. Dixon et al., 1993; Hollis, 2006) |
| *Chironius quadricarinatus* | Semiarboreal | 1067 | 1444 | 377 | 186.2 | Large | (Cacciali & Cabral, 2015; J. Dixon et al., 1993; Hollis, 2006) |
| *Chironius scurrulus* | Arboreal | 2243 | 1515 | 728 | 1117 | Large | (J. Dixon et al., 1993) |
| *Clelia clelia* | Terrestrial | 2240 | 1840 | 400 | 1722.6 | Medium | (Gaiarsa et al., 2013; Scott et al., 2006; G. Scrocchi & Viñas, 1990) |
| *Corallus hortulanus* | Arboreal | 1700 | 1241 | 459 | 3922.3 | Large | (Henderson & Pauers, 2012; Henderson et al., 2013; Pizzatto et al., 2007) |
| *Crotalus durissus* | Terrestrial | 1800 | 1674 | 126 | 2883 | Medium | (Cei, 1993; M. B. Harvey et al., 2005) |
| *Dipsas bucephala* | Arboreal | 571 | 426 | 145 | 49.6 | Large | (M. B. Harvey & Embert, 2008; Hoge & Romano, 1975; Lions et al., 2000; Torello-Viera et al., 2012) |
| *Dipsas catesbyi* | Arboreal | 777 | 542 | 235 | 69.5 | Large | (M. B. Harvey & Embert, 2008) |
| *Dipsas lavillai* | Terrestrial | 534 | 405 | 129 | 36.7 | Large | (Ferreira & Ávila, 2009; G. Scrocchi et al., 1993) |
| *Dipsas mikanii* | Terrestrial | 400 | 322 | 78 | 72.1 | Large | (Braz et al., 2008; Pizzatto, Cantor, et al., 2008; Torello-Viera & Marques, 2017) |
| *Dipsas turgida* | Terrestrial | 515 | 415 | 100 | 48.2 | Large | (Cei, 1993; G. Scrocchi et al., 1993) |
| *Dipsas ventrimaculata* | Terrestrial | 509 | 393 | 116 | 71.2 | Large | (Cei, 1993; G. Scrocchi et al., 1993) |
| *Drymarchon corais* | Semiarboreal | 2406 | 2005 | 401 | 1365.4 | Medium | (Bernarde & Abe, 2006; Cei, 1993; Marcio Martins & Oliveira, 1998) |
| *Epicrates alvarezi* | Semiarboreal | 1613 | 1443 | 170 | 1883.7 | Large | (Cei, 1993; Paulo Passos & Fernandes, 2008; Pizzatto & Marques, 2007) |
| *Epictia albipuncta* | Fossorial | 340 | 316 | 24 | 8 | Small | (Francisco et al., 2012; Kretzschmar, 2006; Laurent, 1984; Pinto et al., 2010) |
| *Epictia australis* | Fossorial | 160 | 153 | 7 | 1.4 | Small | (Cei, 1993; Laurent, 1984; G. Scrocchi, 1990a) |
| *Epictia vellardi* | Fossorial | 155 | 149 | 6 | 2.3 | Small | (Cabral & Netto, 2016; Cei, 1993; Laurent, 1984) |
| *Erythrolamprus aesculapii* | Terrestrial | 1130 | 1005 | 125 | 139.2 | Large | (Curcio et al., 2015; Otávio Augusto Vuolo Marques & Puorto, 1994; Torello-Viera & Marques, 2017) |
| *Erythrolamprus albertguentheri* | Terrestrial | 655 | 545 | 110 | 74.1 | Medium | (Cei, 1993; J. R. Dixon, 1987) |
| *Erythrolamprus almadensis* | Terrestrial | 524 | 392 | 132 | 58.8 | Large | (Cei, 1993; J. R. Dixon, 1987; A. Giraudo, 2002) |
| *Erythrolamprus ceii* | Terrestrial | 524 | 430 | 94 | 32.8 | Medium | (Cei, 1993; J. R. Dixon, 1987) |
| *Erythrolamprus jaegeri* | Aquatic | 539 | 395 | 144 | 62.5 | Large | (Carreira et al., 2005; J. R. Dixon, 1987; A. Giraudo, 2002) |
| *Erythrolamprus miliaris* | Terrestrial | 798 | 644 | 154 | 110 | Medium | (Cei, 1993; J. R. Dixon & Tipton, 2003; Alejandro R. Giraudo et al., 2006; Pizzatto & Marques, 2006) |
| *Erythrolamprus poecilogyrus* | Terrestrial | 793 | 616 | 177 | 68.3 | Medium | (Andrade et al., 2020; Cabral, Bueno-Villafañe, et al., 2017; J. Dixon & Markezich, 1992; Prieto et al., 2012) |
| *Erythrolamprus reginae* | Terrestrial | 960 | 698 | 262 | 98.8 | Large | (Ascenso et al., 2019; Cei, 1993; A. Giraudo, 2002) |
| *Erythrolamprus sagittifer* | Terrestrial | 925 | 648 | 277 | 177.3 | Large | (Cei, 1993; J. Dixon & Thomas, 1982) |
| *Erythrolamprus semiaureus* | Aquatic | 1457 | 1259 | 220 | 168.7 | Medium | (J. R. Dixon, 1983; Alejandro R. Giraudo et al., 2006; Pizzatto, Jordão, et al., 2008; Torello-Viera & Marques, 2017) |
| *Erythrolamprus typhlus* | Terrestrial | 740 | 590 | 150 | 112.7 | Large | (Cei, 1993; J. R. Dixon, 1987) |
| *Eunectes notaeus* | Aquatic | 2550 | 2,180 | 370 | 25204.8 | Medium | (Cacciali, 2009; Cei, 1993; Pizzatto & Marques, 2007; Strussmann & Sazima, 1993) |
| *Helicops angulatus* | Aquatic | 795 | 560 | 235 | 179.6 | Medium | (Henrique C. Costa et al., 2016; Kawashita-Ribeiro et al., 2013; Rossman, 1973) |
| *Helicops infrataeniatus* | Aquatic | 693 | 500 | 193 | 168.7 | Medium | (Cei, 1993; Henrique C. Costa et al., 2016; Kawashita-Ribeiro et al., 2013; Rossman, 1973) |
| *Helicops leopardinus* | Aquatic | 731 | 530 | 201 | 98.8 | Medium | (Cei, 1993; Henrique C. Costa et al., 2016; A. Giraudo, 2002; Kawashita-Ribeiro et al., 2013; Rossman, 1973) |
| *Helicops polylepis* | Aquatic | 1052 | 767 | 285 | 191.8 | Medium | (Henrique C. Costa et al., 2016; Kawashita-Ribeiro et al., 2013; Rossman, 1973) |
| *Hydrodynastes gigas* | Aquatic | 2009 | 1439 | 530 | 2193.6 | Medium | (Cei, 1993; F. Franco et al., 2007; A. Giraudo, 2002) |
| *Hydrops caesurus* | Aquatic | 663 | 570 | 93 | 80 | Small | (Cei, 1993; A. Giraudo, 2002; G. J. Scrocchi et al., 2005) |
| *Imantodes cenchoa* | Arboreal | 1170 | 825 | 345 | 515.8 | Large | (Cei, 1993; de Sousa et al., 2014; Donnelly & Myers, 1991; Pizzatto, Cantor, et al., 2008) |
| *Leptodeira annulata* | Semiarboreal | 655 | 485 | 170 | 185.4 | Large | (Ávila & Morais, 2007; Cei, 1993; Pizzatto, Cantor, et al., 2008) |
| *Leptophis ahaetulla* | Arboreal | 1440 | 956 | 484 | 918.7 | Large | (Cacciali, 2009; Cei, 1993; Nelson R. De Albuquerque et al., 2007, 2012; Nelson Rufino de Albuquerque, 2009; A. Giraudo, 2002) |
| *Liotyphlops beui* | Fossorial | 326 | 321 | 5 | 21 | Small | (Cei, 1993; Centeno et al., 2010; J. R. Dixon & Kofron, 2009; Freire et al., 2007; Parpinelli & Marques, 2008; F. J. M. Santos & Reis, 2018) |
| *Liotyphlops ternetzii* | Fossorial | 413 | 400 | 13 | 18.4 | Small | (Cei, 1993; Centeno et al., 2010; J. R. Dixon & Kofron, 2009; Freire et al., 2007; A. Giraudo, 2002; F. J. M. Santos & Reis, 2018) |
| *Lygophis anomalus* | Terrestrial | 713 | 529 | 184 | 84.1 | Medium | (Carreira et al., 2005; Cei, 1993; J. R. Dixon, 1985) |
| *Lygophis dilepis* | Terrestrial | 690 | 531 | 159 | 44.9 | Medium | (Cacciali, 2009; Cei, 1993) |
| *Lygophis flavifrenatus* | Terrestrial | 746 | 627 | 206 | 82.7 | Medium | (Cacciali, 2009; Carreira, 2002; Carreira et al., 2005; Cei, 1993) |
| *Lygophis meridionalis* | Terrestrial | 598 | 606 | 221 | 96.2 | Medium | (Cacciali, 2009; Cei, 1993) |
| *Lygophis vanzolinii* | Terrestrial | 748 | 545 | 203 | 81.3 | Medium | (Cei, 1993; J. R. Dixon, 1985) |
| *Mastigodryas boddaerti* | Terrestrial | 1800 | 895 | 305 | 331 | Large | (Montingelli et al., 2019; Siqueira et al., 2012) |
| *Micrurus altirostris* | Semifossorial | 783 | 732 | 51 | 483.4 | Small | (N. da Silva, 2016; N. da Silva & Sites, 1999; M. B. Harvey et al., 2003; G. Scrocchi, 1990b) |
| *Micrurus baliocoryphus* | Semifossorial | 1449 | 1374 | 75 | 616.2 | Small | (N. da Silva, 2016; N. da Silva & Sites, 1999; M. B. Harvey et al., 2003; G. Scrocchi, 1990b) |
| *Micrurus diana* | Semifossorial | 1052 | 954 | 54 | 257.2 | Small | (N. da Silva, 2016; N. da Silva & Sites, 1999) |
| *Micrurus frontalis* | Semifossorial | 1794 | 1728 | 66 | 584.9 | Small | (N. da Silva, 2016; N. da Silva & Sites, 1999; G. Scrocchi, 1990b) |
| *Micrurus lemniscatus* | Semifossorial | 1650 | 1550 | 100 | 557.5 | Small | (M. B. Harvey et al., 2003; O. A. V. Marques et al., 2006; Pires et al., 2014) |
| *Micrurus pyrrhocryptus** | Semifossorial | 1747 | 1666 | 81 | 966.5 | Small | (Cei, 1993; N. da Silva, 2016; N. da Silva & Sites, 1999; G. Scrocchi, 1990b) |
| *Micrurus silviae** | Semifossorial | 1113 | 1050 | 63 | 676.1 | Small | (N. da Silva, 2016; Di-Bernardo et al., 2007) |
| *Micrurus surinamensis* | Semifossorial | 1378 | 1198 | 180 | 496.8 | Small | (N. da Silva, 2016; Roze, 1996) |
| *Mussurana bicolor* | Semifossorial | 870 | 710 | 160 | 177.8 | Medium | (Gaiarsa et al., 2013; Gouturier & Faivovich, 1996; Pizzatto, 2005; Scott et al., 2006; G. Scrocchi & Viñas, 1990; H Zaher, 1996) |
| *Mussurana quimi* | Terrestrial | 1090 | 860 | 230 | 352.8 | Medium | (F. Franco et al., 1997; Scott et al., 2006; Silveira & Cotta, 2006) |
| *Oxybelis aeneus* | Arboreal | 1138 | 838 | 300 | 341.7 | Large | (Jadin et al., 2019, 2020, 2021) |
| *Oxyrhopus guibei* | Terrestrial | 955 | 745 | 210 | 354.5 | Large | (Pizzatto, 2005; Torello-Viera & Marques, 2017; Hussam Zaher & Caramaschi, 1992) |
| *Oxyrhopus petolarius* | Terrestrial | 910 | 690 | 220 | 1245.6 | Large | (Cabral & Scott, 2014; Cei, 1993; A. Giraudo, 2002; MacCulloch et al., 2009) |
| *Oxyrhopus rhombifer** | Terrestrial | 712 | 596 | 116 | 129.1 | Large | (Cei, 1993; A. Giraudo, 2002; Torello-Viera & Marques, 2017) |
| *Palusophis_bifossatus* | Terrestrial | 2007 | 1520 | 487 | 450.9 | Large | (Cei, 1993; A. Giraudo, 2002; Leite, 2006; Otavio A.V. Marques & Muriel, 2007; Montingelli et al., 2019) |
| *Paraphimophis rusticus* | Terrestrial | 890 | 800 | 190 | 1406.8 | Medium | (Cei, 1993; Scott et al., 2006; G. Scrocchi & Viñas, 1990; H Zaher, 1996) |
| *Phalotris bilineatus* | Semifossorial | 320 | 290 | 30 | 13.6 | Small | (Cabral & Cacciali, 2015; Carreira et al., 2005; Cei, 1993; A. Giraudo, 2002; Puorto & Ferrarezzi, 1993) |
| *Phalotris lemniscatus* | Semifossorial | 368 | 338 | 30 | 40.5 | Small | (Cabral & Cacciali, 2015; Carreira et al., 2005; Cei, 1993; A. Giraudo, 2002; Puorto & Ferrarezzi, 1993) |
| *Phalotris matogrossensis* | Semifossorial | 495 | 463 | 32 | 30.1 | Small | (Cacciali & Motte, 2007; Lema et al., 2005; Leynaud et al., 2005) |
| *Phalotris sansebastiani* | Semifossorial | 524 | 475 | 49 | 49.4 | Small | (Jansen & Köhler, 2008; G. J. Scrocchi & Giraudo, 2012) |
| *Phalotris tricolor* | Semifossorial | 770 | 725 | 45 | 121.1 | Small | (Cacciali & Motte, 2007; Cei, 1993; Jansen & Köhler, 2008; Lema et al., 2005; Leynaud et al., 2005) |
| *Philodryas aestiva** | Terrestrial | 929 | 628 | 301 | 168.7 | Large | (Carreira et al., 2005; Cei, 1993; Celsi et al., 2008; A. Giraudo, 2002; R. Thomas, 1976) |
| *Philodryas agassizii* | Terrestrial | 307 | 297 | 10 | 29.1 | Medium | (Cei, 1993; Di Pietro et al., 2012; Otavio A.V. Marques et al., 2006) |
| *Philodryas baroni** | Semiarboreal | 1500 | 1211 | 499 | 471.6 | Large | (Briguera et al., 2006; Cacciali, 2009; Cei, 1993) |
| *Philodryas livida** | Terrestrial | 693 | 530 | 163 | 113.8 | Medium | (Smith et al., 2014; Robert Thomas & Fernandes, 1996) |
| *Philodryas mattogrossensis** | Terrestrial | 1522 | 1039 | 483 | 471.6 | Large | (Cacciali et al., 2016; R. Thomas, 1976) |
| *Philodryas olfersii** | Semiarboreal | 1400 | 718 | 354 | 452.7 | Large | (Carreira et al., 2005; Cei, 1993; A. Giraudo, 2002; Hartmann & Marques, 2005; R. Thomas, 1976) |
| *Philodryas patagoniensis** | Terrestrial | 1500 | 860 | 311 | 512.5 | Large | (Cei, 1993; Hartmann & Marques, 2005; López & Giraudo, 2008) |
| *Philodryas psammophidea** | Terrestrial | 1285 | 1015 | 270 | 168.7 | Large | (Cei, 1993; Quinteros-Muñoz et al., 2010; R. Thomas, 1976) |
| *Philodryas trilineata* | Arboreal | 1304 | 908 | 396 | 647.8 | Large | (Cei, 1993; R. Thomas, 1976) |
| *Philodryas varia* | Arboreal | 1151 | 861 | 290 | 361 | Large | (Cei, 1993; R. Thomas, 1976) |
| *Phimophis guerini* | Terrestrial | 1161 | 965 | 196 | 320.2 | Medium | (Cei, 1993; Filho et al., 2012; A. Giraudo, 2002; O. A. V Marques et al., 2001) |
| *Phimophis vittatus* | Semifossorial | 700 | 600 | 100 | 68.3 | Medium | (Alencar et al., 2013; Cei, 1993) |
| *Pseudoboa coronata* | Terrestrial | 723 | 644 | 179 | 203.1 | Medium | (Alencar et al., 2013; Marcio Martins & Oliveira, 1998) |
| *Pseudoboa nigra* | Terrestrial | 1215 | 930 | 285 | 527.1 | Medium | (Cacciali, 2009; Cei, 1993; De Paula Orofno et al., 2010; A. Giraudo, 2002) |
| *Pseudoeryx plicatilis* | Aquatic | 826 | 655 | 171 | 200.2 | Small | (Cabral & Caballero, 2012; Cei, 1993; Scartozzoni et al., 2010) |
| *Pseudotomodon trigonatus* | Terrestrial | 400 | 355 | 45 | 22.3 | Medium | (Cei, 1993; M. B. Harvey & Muñoz, 2004) |
| *Psomophis genimaculatus* | Terrestrial | 387 | 302 | 85 | 22.3 | Large | (O. Marques et al., 2015, 2005; S. Nenda, 2007) |
| *Psomophis obtusus* | Terrestrial | 359 | 300 | 95 | 22.9 | Large | (Cei, 1993; O. Marques et al., 2015) |
| *Rena unguirostris* | Fossorial | 103 | 101 | 2 | 2 | Small | (Cei, 1993; Laurent, 1984; Pinto et al., 2010) |
| *Simophis rhinostoma* | Terrestrial | 887 | 691 | 196 | 112.3 | Medium | (Cacciali et al., 2009; O. Marques et al., 2005) |
| *Siphlophis compressus* | Arboreal | 1420 | 1136 | 284 | 418.5 | Large | (Guedes et al., 2011; Sheehy et al., 2014) |
| *Siphlophis worontzowi* | Arboreal | 1107 | 885 | 222 | 137.4 | Large | (Henrique Caldeira Costa et al., 2010; Dal Vechio et al., 2015; Prudente et al., 2017) |
| *Spilotes pullatus* | Arboreal | 2100 | 1200 | 900 | 3030.1 | Medium | (Cei, 1993; A. Giraudo, 2002; Hauzman et al., 2005) |
| *Taeniophallus occipitalis* | Terrestrial | 504 | 379 | 125 | 40.5 | Large | (C. Myers & Cadle, 1994; C. W. Myers, 1974) |
| *Taeniophallus poecilopogon* | Terrestrial | 390 | 278 | 112 | 21.4 | Large | (Carreira et al., 2005; Cei, 1993; C. Myers & Cadle, 1994; C. W. Myers, 1974) |
| *Tantilla melanocephala* | Semifossorial | 370 | 290 | 80 | 15.4 | Medium | (Burgos Gallardo et al., 2012; Cei, 1993; Sawaya & Sazima, 2003; Wilson, 1999) |
| *Thamnodynastes chaquensis* | Terrestrial | 793 | 619 | 174 | 95.8 | Large | (J. R. Bailey et al., 2005; Bellini et al., 2014; Bergna & Alvarez, 1993) |
| *Thamnodynastes hypoconia* | Aquatic | 730 | 537 | 193 | 56.6 | Large | (J. R. Bailey et al., 2005; Bellini et al., 2014; F. L. Franco et al., 2003) |
| *Thamnodynastes lanei* | Terrestrial | 648 | 479 | 169 | 56.1 | Large | (J. R. Bailey et al., 2005) |
| *Thamnodynastes pallidus* | Terrestrial | 764 | 600 | 164 | 22.3 | Large | (J. R. Bailey et al., 2005; Bellini et al., 2014) |
| *Thamnodynastes strigatus* | Semiarboreal | 860 | 640 | 220 | 95.8 | Large | (J. R. Bailey et al., 2005; Bellini et al., 2014; Cei, 1993) |
| *Tomodon dorsatum* | Terrestrial | 707 | 532 | 175 | 94.7 | Medium | (Bizerra et al., 2005; Cei, 1993; M. B. Harvey & Muñoz, 2004) |
| *Tomodon ocellatus* | Terrestrial | 377 | 334 | 43 | 35.3 | Medium | (Cei, 1993; M. B. Harvey & Muñoz, 2004) |
| *Tomodon orestes* | Terrestrial | 515 | 448 | 67 | 31.3 | Medium | (M. B. Harvey & Muñoz, 2004) |
| *Xenodon dorbignyi* | Semifossorial | 443 | 372 | 71 | 95.8 | Medium | (Carreira et al., 2005; Cei, 1993; S. J. Nenda & Cacivio, 2007; Tozetti et al., 2009; Yanosky & Chani, 1988) |
| *Xenodon histricus* | Semifossorial | 333 | 282 | 51 | 11.3 | Medium | (Alves et al., 2013; Carreira et al., 2005; Cei, 1993) |
| *Xenodon matogrossensis** | Semifossorial | 462 | 395 | 67 | 21.1 | Medium | (Cabral et al., 2020) |
| *Xenodon merremi* | Terrestrial | 783 | 656 | 127 | 168.7 | Medium | (Cacciali, 2010; Pizzatto, Jordão, et al., 2008) |
| *Xenodon pulcher** | Semifossorial | 510 | 440 | 70 | 68.3 | Medium | (Cei, 1993; S. J. Nenda & Cacivio, 2007; G. Scrocchi & Cruz, 1993) |
| *Xenodon semicinctus* | Semifossorial | 412 | 366 | 46 | 46.2 | Medium | (Cei, 1993; S. J. Nenda & Cacivio, 2007; G. Scrocchi & Cruz, 1993) |
| *Xenodon severus* | Terrestrial | 1000 | 840 | 160 | 978.2 | Medium | (Chippaux, 1986; Kahn, 2010; M. V. Silva et al., 2006) |
| *Xenopholis undulatus* | Terrestrial | 465 | 395 | 70 | 24.2 | Medium | (Henrique Caldeira Costa et al., 2013; Gomes et al., 2020; Jansen et al., 2009) |

Adalsteinsson, S. A., Branch, W. R., Trape, S., Vitt, L. J., & Hedges, S. B. (2009). Molecular phylogeny, classification, and biogeography of snakes of the family Leptotyphlopidae (Reptilia, Squamata). *Zootaxa*, *50*(2244), 1–50. https://doi.org/10.11646/zootaxa.2244.1.1

Alencar, L. R. V., Gaiarsa, M. P., & Martins, M. (2013). The Evolution of Diet and Microhabitat use in Pseudoboine Snakes. *South American Journal of Herpetology*, *8*(1), 60–66. https://doi.org/10.2994/sajh-d-13-00005.1

Almeida, P. C., Feitosa, D. T., Passos, P., & Prudente, A. L. C. (2014). Morphological variation and taxonomy of Atractus latifrons (Günther, 1868) (Serpentes: Dipsadidae). *Zootaxa*, *3860*(1), 64–80. https://doi.org/10.11646/zootaxa.3860.1.3

Almeida-Santos, S. M., & Marques, O. A. V. (2002). Male-male ritual combat in the colubrid snake Chironius bicarinatus from the Atlantic Forest, southeastern Brazil. *Amphibia Reptilia*, Vol. 23, pp. 528–533.

Alves, S. da S., Bolzan, A. M. R., do Santos, T. G., Gressler, D. T., & Cechin, S. Z. (2013). Rediscovery, distribution extension and defensive behaviour of Xenodon histricus (Squamata: Serpentes) in the state of Rio Grande do Sul, Brazil. *Salamandra*, *49*(4), 219–222.

Andrade, H., Costa, S., Santos, M., & Dias, E. (2020). Diet review of Erythrolamprus poecilogyrus (Wied-Neuwied, 1825) (Serpentes: Dipsadidae), and first record of Dermatonotus muelleri (Boettger, 1885) (Anura: Microhylidae) as a prey item in Sergipe State, northeastern Brazil. *Herpetology Notes*, *13*, 10651068.

Ascenso, A. C., Costa, J. C. L., & Prudente, A. L. C. (2019). Taxonomic revision of the Erythrolamprus reginae species group, with description of a new species from Guiana Shield (Serpentes: Xenodontinae). In *Zootaxa* (Vol. 4586). https://doi.org/10.11646/zootaxa.4586.1.3

Avila, R. W., Ferreira, V. L., & Souza, V. B. (2006). Biology of the blindsnake Typhlops brongersmianus (Typhlopidae) in a semideciduous forest from Central Brazil. *Herpetological Journal*, *16*(4), 403–405.

Ávila, R. W., & Morais, D. H. (2007). Notes on the ecology of the colubrid snake Leptodeira annulata in the Pantanal, Brazil. *Herpetological Review*, *38*(3), 278–280.

Bailey, J. R., Thomas, R. A., & da Silva, N. J. (2005). A revision of the South American snake genus Thamnodynastes Wagler, 1830 (Serpentes, Colubridae, Tachymenini). I. Two new species of Thamnodynastes from Central Brazil and adjacent areas, with a redefinition of and neotype designation for Thamnodynastes p. *Phyllomedusa*, *4*(2), 83–101. https://doi.org/10.11606/issn.2316-9079.v4i2p83-101

Bailey, R. J. (1955). The snakes of the genus Chironius in southeastern South America. *Occasional Papers of the Museum of Zoology, University of Michigan*, *571*(571), 1–21. https://doi.org/10.1038/469481a

Balestrin, R. L., & Di-Bernardo, M. (2005). Reproductive biology of Atractus reticulatus (Boulenger, 1885) (Serpentes, Colubridae) in southern Brazil. *Herpetological Journal*, *15*(3), 195–199.

Bellini, G. P., Giraudo, A. R., & Arzamendia, V. (2014). Comparative ecology of three species of Thamnodynastes (Serpentes, Dipsadidae) in subtropical-temperate South America. *Herpetological Journal*, *24*(2), 87–96.

Bergna, S., & Alvarez, B. (1993). Descripción de una nueva especie de Thamnodynastes (Reptilia: Serpentes, Colubridae) del nordeste argentina. *FACENA*, *10*, 5–18.

Bernarde, P. S., & Abe, A. S. (2006). A Snake Community At Espigão Do Oeste, Rondônia, Southwestern Amazon, Brazil. *South American Journal of Herpetology*, *1*(2), 102–113. https://doi.org/10.2994/1808-9798(2006)1[102:ascaed]2.0.co;2

Bertona, M., & Chiaraviglio, M. (2003). Reproductive biology, mating aggregations, and sexual dimorphism of the Argentine Boa Constrictor (Boa constrictor occidentalis). *Journal of Herpetology*, *37*(3), 510–516. https://doi.org/10.1670/122-02A

Bizerra, A., Marques, O. A. V., & Sazima, I. (2005). Reproduction and feeding of the colubrid snake Tomodon dorsatus from south-eastern Brazil. *Amphibia Reptilia*, *26*(1), 33–38. https://doi.org/10.1163/1568538053693350

Boback, S. M. (2005). Natural history and conservation of island boas (Boa constrictor) in Belize. *Copeia*, *2005*(4), 880–885. https://doi.org/10.1643/0045-8511(2005)005[0880:NHACOI]2.0.CO;2

Boback, S. M. (2006). A morphometric comparison of island and mainland boas (Boa constrictor) in Belize. *Copeia*, *2006*(2), 261–267. https://doi.org/10.1643/0045-8511(2006)6[261:AMCOIA]2.0.CO;2

Braz, H. B. P., Franco, F. L., & Almeida-Santos, S. M. (2008). Communal egg-laying and nest-sites of the Goo-eater Snake, Sibynomorphus mikanii (Dipsadidae, Dipsadinae) in southeastern Brazil. *Herpetological Bulletin*, (106), 26–30.

Briguera, V., Tamburini, D., Kufner, M. B., Gavier, G., Giraudo, L., Torres, R., & Bechara, V. (2006). Herpetofauna en relictos de Bosque Chaqueño de la región de Mar Chiquita, Córdoba. *Cuadernos de Herpetología*, *20*(1), 25–31.

Burbrink, F. T., & Myers, E. A. (2015). Both traits and phylogenetic history influence community structure in snakes over steep environmental gradients. *Ecography*, *38*(10), 1036–1048. https://doi.org/10.1111/ecog.01148

Burgos Gallardo, F., Ramos, B., & Baldo, J. L. (2012). Tantilla melanocephala (Linnaeus, 1758) - (Serpentes: Colubridae). Primeros registros para la Provincia de Jujuy y confirmación de su presencia en el noroeste argentino. *Cuadernos de Herpetología*, *26*(2), 96–98. https://doi.org/10.31017/1538

Cabral, H. (2014). Distribución del género Atractus (Wagler, 1828) (Serpentes, Dipsadidae) en Paraguay, con comentarios sobre sus afinidades ecológicas. *Paraquaria Natural*, *2*(2), 61–65.

Cabral, H., Bueno-Villafañe, D., & Romero-Nardelli, L. (2017). Comments on the diet of juvenile Erythrolamprus poecilogyrus caesius (Serpentes: Dipsadidae) in the Paraguayan Chaco. *Phyllomedusa*, *16*(2), 299–302. https://doi.org/10.11606/issn.2316-9079.vl6i2p299-302

Cabral, H., & Caballero, A. (2012). Pseudoeryx plicatilis. Distribution. *Herpetological Review*, *43*(4), 622.

Cabral, H., & Cacciali, P. (2015). A new species of phalotris (Serpentes: Dipsadidae) from the paraguayan chaco. *Herpetologica*, *71*(1), 72–77. https://doi.org/10.1655/HERPETOLOGICA-D-14-00021

Cabral, H., De Lema, T., & Renner, M. F. (2017). Revalidation of Apostolepis barrioi (Serpentes: Dipsadidae). *Phyllomedusa*, *16*(2), 243–254. https://doi.org/10.11606/issn.2316-9079.vl6i2p243-254

Cabral, H., & Netto, F. (2016). Epictia vellardi. Distribution. *Herpetological Review*, *47*(1), 83.

Cabral, H., & Perez, N. (2015). Apostolepis assimilis (Reinhardt, 1861) (Serpentes: Dipsadidae): Segundo registro para Paraguay. *Boletin Del Museo Nacional de Historia Natural Del Paraguay*, *19*(1), 19–22.

Cabral, H., Piatti, L., Martins, M., & Ferreira, V. L. (2020). *Natural history of Xenodon matogrossensis ( Scrocchi and Cruz , 1993 ) ( Serpentes , Dipsadidae ) in the Brazilian Pantanal*. *34*(2), 1–8. https://doi.org/10.31017/CdH.2020.(2020-026)ABSTRACT

Cabral, H., Rojas, V., Galluppi, T., Ortiz, E., & Baez, M. (2017). Comments on the diet of Bothrops alternatus Duméril, Bibron& Duméril,1854. *Herpetology Notes*, *10*(April), 219–220.

Cabral, H., & Scott, N. J. (2014). Oxyrhopus petolarius (Linnaeus, 1758) (Serpentes, Dipsadidae): Distribution extension and new departmental record for Paraguay. *Check List*, *10*(5), 1207–1209. https://doi.org/10.15560/10.5.1207

Cacciali, P. (2009). *del Guía para la identificación de 60 Serpientes del Paraguay*. Asunción: Guyra Paraguay.

Cacciali, P. (2010). Chromatic variation in populations of Xenodon merremi (Serpentes : Dipsadidae) in Paraguay. *Acta Herpetologica*, *5*(1), 107–112.

Cacciali, P., & Cabral, H. (2015). The genus Chironius (Serpentes, Colubridae) in Paraguay : composition, distribution, and morphology. *Basic and Applied Herpetology*, *29*, 51–60.

Cacciali, P., Cabral, H., Ferreira, V. L., & Köhler, G. (2016). Revision of Philodryas mattogrossensis with the revalidation of P. erlandi (Reptilia: Squamata: Dipsadidae). *Salamandra*, *52*(4), 293–305.

Cacciali, P., & Motte, M. (2007). Variación intraespecífica en Phalotris matogrossensis y P. tricolor: Una evaluación de sus caracteres diagnósticos (Squamata, Colubridae). *Cuadernos de Herpetologia*, *21*(2), 75–82.

Cacciali, P., Scott, N., Guenther, R., Sawaya, R., Brusquetti, F., & Bauer, F. (2009). Taxonomic Status of the False Coral Snake Genus Simophis (Peters, 1860) (Serpentes: Colubridae: Colubrinae) from Paraguay and Brazil. *Journal of Herpetology*, *43*(4), 698–703.

Carrasco, P. A., Leynaud, G. C., & Scrocchi, G. J. (2010). Redescription of the southernmost snake species, Bothrops ammodytoides (Serpentes: Viperidae: Crotalinae). *Amphibia Reptilia*, *31*(3), 323–338. https://doi.org/10.1163/156853810791769491

Carreira, S. (2002). Alimentación de los ofidios de Uruguay. In *Monografías de Herpetología* (Vol. 6). Asociación Herpetológica Española.

Carreira, S., Meneghel, M., & Achaval, F. (2005). *Reptiles de Uruguay*. Montevideo: Universidad de la Republica.

Cei, J. (1993). Reptiles del noroeste, nordeste y este de la Argentina. *Museo Regionale Sci. Naturale Torino, Monografie*, *14*, 1–949.

Celsi, C. E., Monserrat, A. L., & Kacoliris, F. P. (2008). Reptilia, Colubridae, Philodryas aestivus: distribution extension. *Check List*, *4*(1), 12. https://doi.org/10.15560/4.1.12

Centeno, F. C., Sawaya, R. J., & Germano, V. J. (2010). A new species of Liotyphlops (Serpentes: Anomalepididae) from the Atlantic Coastal Forest in Southeastern Brazil. *Herpetologica*, *66*(1), 86–91. https://doi.org/10.1655/08-079.1

Chippaux, J.-P. (1986). Les serpents de la guyane française. In *Toxicon* (Vol. 26). https://doi.org/10.1016/0041-0101(88)90263-2

Costa, Henrique C., Santana, D. J., Leal, F., Koroiva, R., & Garcia, P. C. A. (2016). A New Species of Helicops (Serpentes: Dipsadidae: Hydropsini) from Southeastern Brazil. *Herpetologica*, *72*(2), 157–166. https://doi.org/10.1655/HERPETOLOGICA-D-15-00059

Costa, Henrique Caldeira, Clara, M., & Gurgel, C. (2013). Xenopholis undulatus ( Serpentes : Xenodontinae ): Reprodução e alimentação em cativeiro. *Herpetologia Brasileira*, *2*(April 2014), 36–38.

Costa, Henrique Caldeira, São-Pedro, V. de A., & Feio, R. N. (2010). A new record of the poorly known Amazonian snake Siphlophis worontzowi. *Herpetology Notes*, *3*(1), 97–100.

Curcio, F. F., Scali, S., & Rodrigues, M. T. (2015). Taxonomic Status of Erythrolamprus bizona Jan (1863) (Serpentes, Xenodontinae): Assembling a Puzzle with Many Missing Pieces. *Herpetological Monographs*, *29*(1), 40–64. https://doi.org/10.1655/HERPMONOGRAPHS-D-15-00002

da Silva, N. (2016). *As Cobras-Corais do Brasil - Biologia, Taxonomia, Venenos e Envenenamentos*. Goiás: PUC - Goiás.

da Silva, N., & Sites, J. (1999). Revision of the Micrurus frontalis Complex (Serpentes: Elapidae). *Herpetological Monographs*, *13*, 142–194.

da Silva, V. X., & Rodrigues, M. T. (2008). Taxonomic revision of the Bothrops neuwiedi complex (Serpentes, Viperidae) with description of a new species. *Phyllomedusa*, *7*(1), 45–90. https://doi.org/10.11606/issn.2316-9079.v7i1p45-90

Dal Vechio, F., Teixeira Junior, M., Neto, A. M., & Rodrigues, M. T. (2015). On the snake Siphlophis worontzowi (Prado, 1940): Notes on its distribution, diet and morphological data. *Check List*, *11*(1), 1–5. https://doi.org/10.15560/11.1.1534

Daru, B. H., Karunarathne, P., & Schliep, K. (2020). phyloregion: R package for biogeographical regionalization and macroecology. *Methods in Ecology and Evolution*, *11*(11), 1483–1491. https://doi.org/10.1111/2041-210X.13478

De Albuquerque, Nelson R., Galatti, U., & Di-Bernardo, M. (2007). Diet and feeding behaviour of the Neotropical parrot snake (Leptophis ahaetulla) in northern Brazil. *Journal of Natural History*, *41*(17–20), 1237–1243. https://doi.org/10.1080/00222930701400954

De Albuquerque, Nelson R., Passos, P., & Gotte, S. W. (2012). Leptophis santamartensis (Serpentes, Colubridae), a junior synonym of Leptophis ahaetulla occidentalis. *Journal of Herpetology*, *46*(2), 248–252. https://doi.org/10.1670/10-263

de Albuquerque, Nelson Rufino. (2009). New records of Leptophis ahaetulla ahaetulla (Serpentes, Colubridae) for Venezuela, Colombia and the placement of L. a. copei into the synonymy of L. a. ahaetulla. *Biota Neotropica*, *9*(4), 293–296. https://doi.org/10.1590/s1676-06032009000400035

De Albuquerque, Nelson Rufino, & De Lema, T. (2012). Description of the second known specimen of Apostolepis intermedia (Serpentes, Colubridae, Xenodontinae). *Zootaxa*, *58*(3325), 53–58. https://doi.org/10.11646/zootaxa.3325.1.4

de Lema, T. (2001). Fossorial snake genus Apostolepis from South America (Serpentes: Colubridae: Elapomorphinae). *Cuadernos de Herpetología*, *15*(1), 29–43.

De Paula Orofno, R., Pizzatto, L., & Marques, O. A. V. (2010). Reproductive biology and food habits of Pseudoboa nigra (Serpentes: Dipsadidae) from the Brazilian cerrado. *Phyllomedusa*, *9*(1), 53–61. https://doi.org/10.11606/issn.2316-9079.v9i1p53-61

de Sousa, K. R. M., Prudente, A. L. C., & Maschio, G. F. (2014). Reproduction and diet of Imantodes cenchoa (Dipsadidae: Dipsadinae) from the Brazilian Amazon. *Zoologia*, *31*(1), 8–19. https://doi.org/10.1590/S1984-46702014000100002

de Souza Filho, G. A., de Moura-Leite, J. C., Matias, E. G., & Morato, S. A. A. (2012). Chironius fuscus (Linnaeus, 1758) (Serpentes: Colubridae): Distribution extension, new state record and variation in southern Brazil. *Check List*, *8*(6), 1315–1318. https://doi.org/10.15560/8.6.1315

Di Pietro, D. O., Christie, M., & Williams, J. (2012). Nuevos registros de Philodryas agassizii (Serpentes: Dipsadidae: Xenodontinae) en la Argentina. *Cuadernos de Herpetología*, *27*(1), 0–0. https://doi.org/10.31017/1506

Di-Bernardo, M., Borges-Martins, M., & Da Silva, N. J. (2007). A new species of coralsnake (Micrurus: Elapidae) from southern Brazil. *Zootaxa*, *26*(1447), 1–26. https://doi.org/10.11646/zootaxa.1447.1.1

Dixon, J., & Markezich, A. (1992). Taxonomy and geographic variation of Liophis poecilogyrus (Wied) from South America (Serpentes: Cokubridae). *The Texas Journal of Science*, *44*, 131–166.

Dixon, J. R. (1983). Taxonomic Status of the South American Snakes Liophis miliaris, L. amazonicus, L. chrysostomus, L. mossoroensis and L. purpurans (Colubridae: Serpentes). *Copeia*, *1983*(3), 791. https://doi.org/10.2307/1444348

Dixon, J. R. (1985). A Review of Liophis anomalus and Liophis elegantissimus, and the Description of a New Species (Serpentes: Colubridae). *Copeia*, *1985*(3), 565. https://doi.org/10.2307/1444745

Dixon, J. R. (1987). Taxonomy and geographic variation of Liophis typhlus and related “green” species of South America (Serpentes: Colubridae). *Annals of Carnegie Museum*, *56*(8), 173–191.

Dixon, J. R., & Hendricks, F. S. (1979). The Wormsnakes (Family Typhlopidae) of the Neotropics, Exclusive of the Antilles. *Zoologische Verhandelingen*, *173*, 3–39.

Dixon, J. R., & Kofron, C. P. (2009). The Central and South American Anomalepid Snakes of the Genus Liotyphlops. *Amphibia-Reptilia*, *4*(2), 241–264. https://doi.org/10.1163/156853883x00120

Dixon, J. R., & Tipton, B. L. (2003). Liophis miliaris intermedius (Henle and Ehrl, 1991) is actually Liophis reginae (Serpentes: Colubridae). *Journal of Herpetology*, *37*(1), 191. https://doi.org/10.1670/0022-1511(2003)037[0191:LMIHAE]2.0.CO;2

Dixon, J., & Thomas, R. (1982). The status of the Argentine colubrid snakes Liophis sagittifer and L. trifasciatus. *Herpetologica*, *38*(3), 389–395.

Dixon, J., Wiest, J., & Cei, J. (1993). Revision of the Neotropical snake genus Chironius Fitzinger (Serpentes, Colubridae). *Museo Regionale Di Scienze Naturali, Monografie*, *Xiii*, 140.

Donnelly, M. A., & Myers, C. W. (1991). Herpetological Results of the 1990 Venezuelan Expedition to the Summit of Cerro Guaiquinima, with New Tepui Reptiles. *American Museum Novitates*, (3017), 54.

Dos Santos, F. M., Entiauspe-Neto, O. M., Araújo, J. da S., De Souza, M. B., De Lema, T., Strüssmann, C., & De Albuquerque, N. R. (2018). A new species of burrowing snake (Serpentes: Dipsadidae: Apostolepis) from the state of Mato grosso, central-west region of Brazil. *Zoologia*, *35*(1887), 1–10. https://doi.org/10.3897/zoologia.35.e26742

Embert, Dirk., & Reichle, S. (2003). Neue Daten zu Apostolepis multicincta HARVEY, 1999 aus den innerandinen Trockentälern Boliviens. *Salamandra*, *39*(3), 249–253.

Entiauspe-Neto, O. M., de Lema, T., & Cabral, H. (2014). Apostolepis intermedia Koslowsky, 1898 (Serpentes: Xenodontinae: Elapomorphini): First records for Paraguay. *Check List*, *10*(3), 600–601. https://doi.org/10.15560/10.3.600

Faith, D. (1992). Conservation evaluation and phylogenetic diversity. *Biological Conservation*, *61*, 1–10. https://doi.org/10.1016/0003-2697(75)90168-2

Feldman, A., Sabath, N., Pyron, R. A., Mayrose, I., & Meiri, S. (2016). Body sizes and diversification rates of lizards, snakes, amphisbaenians and the tuatara. *Global Ecology and Biogeography*, *25*(2), 187–197. https://doi.org/10.1111/geb.12398

Ferreira, V. L., & Ávila, R. W. (2009). Reptilia, Squamata, Serpentes, Dipsadidae, Sibynomorphus lavillai Scrocchi, Porto and Rey 1993: new country record and geographic distribution map. *Check List*, *5*(4), 773. https://doi.org/10.15560/5.4.773

Filho, G. A. P., Santana, G. G., Vieira, W. L. da S., Alves, R. R. da N., Montenegro, P. F. G. P., & de Freitas, M. A. (2012). Phimophis guerini (Duméril, Bibron and Dumeril, 1854) (Serpentes: Dipsadidae): Distribution extension in Paraiba, Brazil. *Check List*, *8*(5), 966–967. https://doi.org/10.15560/8.5.966

França, D. P. F., Barbo, F. E., Silva, N. J., Silva, H. L. R., & Zaher, H. (2018). A new species of Apostolepis (Serpentes, Dipsadidae, Elapomorphini) from the Cerrado of Central Brazil. *Zootaxa*, *4521*(4), 539–552. https://doi.org/10.11646/zootaxa.4521.4.3

Francisco, B. C. S., Pinto, R. R., & Fernandes, D. S. (2012). Taxonomy of Epictia munoai (Orejas-Miranda, 1961) (Squamata: Serpentes: Leptotyphlopidae). *Zootaxa*, *52*(3512), 42–52. https://doi.org/10.11646/zootaxa.3512.1.2

Franco, F., Fernandes, D., & Bentim, B. (2007). A new species of Hydrodynastes Fitzinger, 1843 from central Brazil (Serpentes: Colubridae: Xenodontinae). *Zootaxa*, *1613*, 57–65. https://doi.org/10.5281/zenodo.179001

Franco, F. L., Ferreira, T. G., Marques, O. A. v., & Sazima, I. (2003). A new specie of hood-displaying Thamnodynastes (Serpentes: Colubridae) from the Atlantic forest in southeast Brazil. *Zootaxa*, *334*, 1–7.

Franco, F., Marques, O., & Puorto, G. (1997). Two new species of colubrid snakes of the Genus Clelia from Brazil. *Journal of Herpetology*, *31*(4), 483–490.

Freire, E., Caramashi, U., & Argolo, A. (2007). A new species of Liotyphlops (Serpentes: Anomalepididae) from the Atlantic Rain Forest of Northeastern Brazil. *Zootaxa*, *1393*, 19–26. https://doi.org/10.11646/zootaxa.2719.1.5

Gaiarsa, M. P., de Alencar, L. R. V., & Martins, M. (2013). Natural history of pseudoboine snakes. *Papeis Avulsos de Zoologia*, *53*(19), 261–283. https://doi.org/10.1590/S0031-10492013001900001

Giraudo, A. (2002). *Serpientes de la Selva Paranaense y del Chaco Húmedo*. Buenos Aires: Literature of Latin America.

Giraudo, A. R., & Scrocchi, G. J. (2000). The genus atractus (serpentes: Colubridae) in north-eastern Argentina. *Herpetological Journal*, Vol. 10, pp. 81–90.

Giraudo, Alejandro R., Arzamendia, V., & Cacciali, P. (2006). Geographic variation and taxonomic status of the southernmost populations of Liophis miliaris (Linnaeus, 1758) (Serpentes: Colubridae). *Herpetological Journal*, *16*(2), 213–220.

Giraudo, Alejandro R., & Scrocchi, G. J. (1998). A new species of Apostolepis (Serpentes: Colubridae) and comments on the genus in Argentina. *Herpetologica*, *54*(4), 470–476.

Gomes, D. F., Azevedo, J., Murta-Fonseca, R., Faurby, S., Antonelli, A., & Passos, P. (2020). Taxonomic revision of the genus Xenopholis Peters, 1869 (Serpentes: Dipsadidae): Integrating morphology with ecological niche. In *PLoS ONE* (Vol. 15). https://doi.org/10.1371/journal.pone.0243210

Gouturier, G., & Faivovich, J. (1996). Clelia bicolor (Peracca) en la Provincia de Santa Fe. *Cuadernos de Herpetologia*, *10*, 1–2.

Graboski, R., Arredondo, J. C., Grazziotin, F. G., da Silva, A. A. A., Prudente, A. L. C., Rodrigues, M. T., … Zaher, H. (2019). Molecular phylogeny and hemipenial diversity of South American species of Amerotyphlops (Typhlopidae, Scolecophidia). *Zoologica Scripta*, *48*(2), 139–156. https://doi.org/10.1111/zsc.12334

Graham, C. H., & Fine, P. V. A. (2008). Phylogenetic beta diversity: Linking ecological and evolutionary processes across space in time. *Ecology Letters*, *11*(12), 1265–1277. https://doi.org/10.1111/j.1461-0248.2008.01256.x

Guedes, T. B., Nunes, G. S. S., Lúcia, A., & Marques, O. A. V. (2011). New records and geographical distribution of the Tropical Banded Treesnake Siphlophis compressus (Dipsadidae) in Brazil. *Herpetology Notes*, *4*(October), 341–346.

Hamdan, B., & Fernandes, D. S. (2015). Taxonomic revision of Chironius flavolineatus (Jan, 1863) with description of a new species (Serpentes: Colubridae). *Zootaxa*, *4012*(1), 97–119. https://doi.org/10.11646/zootaxa.4012.1.5

Hamdan, B., Scali, S., & Fernandes, D. S. (2014). On the identity of Chironius flavolineatus (Serpentes: Colubridae). *Zootaxa*, *3794*(1), 134–142. https://doi.org/10.11646/zootaxa.3794.1.6

Hartmann, P. A., & Marques, O. A. V. (2005). Diet and habitat use of two sympatric species of Philodryas (Colubridae), in south Brazil. *Amphibia Reptilia*, *26*(1), 25–31. https://doi.org/10.1163/1568538053693251

Harvey, M. (1999). Revision of Bolivian Apostolepis (Squamata: Colubridae). *Copeia*, *2*, 388–409.

Harvey, M. B. (2008). New and poorly known Dipsas (Serpentes: Colubridae) from Northern South America. *Herpetologica*, *64*(4), 422–451. https://doi.org/10.1655/07-068R1.1

Harvey, M. B., A, L. G., Scrocchi, G. J., Copeia, S., & Gonzales, L. (2008). New Species of Apostolepis (Squamata: Colubridae) from the Gran Chaco in Southern Bolivia. *Copeia*, *2001*(2), 501–507.

Harvey, M. B., Aparicio E., J., & Gonzalez A., L. (2003). Revision of the venomous snakes of Bolivia: Part 1. The coralsnakes (Elapidae: Micrurus). *Annals of Carnegie Museum*, *72*(1), 1–52.

Harvey, M. B., Aparicio, J. E., & Gonzales, L. A. (2005). Revision of the venomous snakes of Bolivia. II: The pitvipers (Serpentes: Viperidae). *Annals of Carnegie Museum*, *74*(1), 1–37. https://doi.org/10.2992/0097-4463(2005)74[1:ROTVSO]2.0.CO;2

Harvey, M. B., & Embert, D. (2008). Review of bolivian Dipsas (Serpentes: Colubridae), with comments on other South American species. *Herpetological Monographs*, (22), 54–105. https://doi.org/10.1655/07-023.1

Harvey, M. B., & Muñoz, A. (2004). A new species of Tomodon (Serpentes: Colubridae) from high elevations in the Bolivian Andes. *Herpetologica*, *60*(3), 364–372. https://doi.org/10.1655/03-57

Hauzman, E., Costa, A., & Scartozzoni, R. (2005). Spilotes pullatus - Reproduction. *Herpetological Review*, *36*(3), 328.

Hedges, S. B., Marion, A. B., Lipp, K. M., Marin, J., & Vidal, N. (2014). A taxonomic framework for typhlopid snakes from the Caribbean and other regions (Reptilia, Squamata). *Caribbean Herpetology*, 1–61. https://doi.org/10.31611/ch.49

Helmus, M. R., Bland, T. J., Williams, C. K., & Ives, A. R. (2007). Phylogenetic measures of biodiversity. *The American Naturalist*, *169*(3). https://doi.org/10.1086/511334

Henderson, R. W., & Pauers, M. J. (2012). On the Diets of Neotropical Treeboas (Squamata: Boidae: Corallus ) 1 . *South American Journal of Herpetology*, *7*(2), 172–180. https://doi.org/10.2994/057.007.0207

Henderson, R. W., Pauers, M. J., & Colston, T. J. (2013). On the congruence of morphology, trophic ecology, and phylogeny in Neotropical treeboas (Squamata: Boidae: Corallus). *Biological Journal of the Linnean Society*, *109*(2), 466–475. https://doi.org/10.1111/bij.12052

Hoge, A. R., & Romano, S. A. de L. (1975). A New Subspecies of Dipsas indica from Brazil (Serpentes, Colubridae, Dipsadinae). *Mem. Inst. Butantan*, pp. 51–60.

Hollis, J. L. (2006). Phylogenetics of the genus Chironius fitzinger, 1826 (Serpentes, Colubridae) based on morphology. *Herpetologica*, *62*(4), 435–453. https://doi.org/10.1655/0018-0831(2006)62[435:POTGCF]2.0.CO;2

Hoogmoed, M. S. (1980). Revision of the genus Atractus in Surinam, with the resurrection of two species (Colubridae, Reptilia). Notes on the herpetofauna of Surinam VII. *Zoologische Verhandelingen*, (175), 1–47.

Jadin, R. C., Blair, C., Jowers, M. J., Carmona, A., & Murphy, J. C. (2019). Hiding in the lianas of the tree of life: Molecular phylogenetics and species delimitation reveal considerable cryptic diversity of New World Vine Snakes. *Molecular Phylogenetics and Evolution*, *134*(December 2018), 61–65. https://doi.org/10.1016/j.ympev.2019.01.022

Jadin, R. C., Blair, C., Orlofske, S. A., Jowers, M. J., Rivas, G. A., Vitt, L. J., … Murphy, J. C. (2020). Not withering on the evolutionary vine: systematic revision of the Brown Vine Snake (Reptilia: Squamata: Oxybelis) from its northern distribution. *Organisms Diversity and Evolution*, *20*(4), 723–746. https://doi.org/10.1007/s13127-020-00461-0

Jadin, R. C., Jowers, M. J., Orlofske, S. A., Duellman, W. E., Blair, C., & Murphy, J. C. (2021). A new vine snake (Reptilia, Colubridae, Oxybelis) from Peru and redescription of O. acuminatus. *Evolutionary Systematics*, *5*(1), 1–12. https://doi.org/10.3897/evolsyst.5.60626

Jansen, M., Álvarez, L. G., & KÖhler, G. (2009). Description of a new species of Xenopholis (Serpentes: Colubridae) from the cerrado of bolivia, with comments on xenopholis scalaris in Bolivia. *Zootaxa*, (2222), 31–45. https://doi.org/10.11646/zootaxa.2222.1.3

Jansen, M., & Köhler, G. (2008). A new species of Phalotris from the eastern lowlands of Bolivia. *Senckenbergiana Biologica*, *88*, 103–110.

Kahn, T. (2010). Cobra-like hooding and mouth-gapping in an atypically monocle patterned Xenodon severus (Linnaeus, 1758): a case of convergent evolutionary behavior? *Herpetotropicos*, *6*(1), 25–26.

Kawashita-Ribeiro, R., Ávila, R., & Morais, D. (2013). A NEW SNAKE OF THE GENUS HELICOPS WAGLER, 1830 (DIPSADIDAE, XENODONTINAE) FROM BRAZIL. *Herpetologica*, *69*(1), 80–90.

Kretzschmar, S. (2006). Revisión histórica y redescripción de Leptotyphlops albipunctus (Serpentes: Leptotyphlopidae). *Cuadernos de Herpetología*, *19*(2), 43–56.

Laffan, S. W., Rosauer, D. F., Di Virgilio, G., Miller, J. T., González-Orozco, C. E., Knerr, N., … Mishler, B. D. (2016). Range-weighted metrics of species and phylogenetic turnover can better resolve biogeographic transition zones. *Methods in Ecology and Evolution*, *7*(5), 580–588. https://doi.org/10.1111/2041-210X.12513

Laurent, R. (1984). El genero Leptotyphlops en la colección de la Fundación Miguel Lillo. *Acta Zoologica Lilloana*, *38*(1), 29–34.

Leão, S. M., Pelegrin, N., de Campos Nogueira, C., & Brandão, R. A. (2014). Natural history of Bothrops itapetiningae boulenger, 1907 (serpentes: Viperidae: crotalinae), an endemic species of the Brazilian Cerrado. *Journal of Herpetology*, *48*(3), 324–331. https://doi.org/10.1670/12-191

Leite, P. T. (2006). *História Natural de Mastigodryas bifossatus (Serpentes, Colubridae) em Domínio Subtropical no Brasil*.

Lema, T. de, D’Agostini, F. M., & Cappellari, L. H. (2005). Nova espécie de Phalotris, redescrição de P. tricolor e osteologia craniana (Serpentes, Elapomorphinae). *Iheringia. Série Zoologia*, *95*(1), 65–78. https://doi.org/10.1590/s0073-47212005000100010

Leynaud, G. C., Cabrera, M. R., & Carrasco, P. (2005). A survey of the southernmost representatives of the tricolor species group, genus Phalotris (Serpentes, Colubridae). *Phyllomedusa*, *4*(2), 103–110. https://doi.org/10.11606/issn.2316-9079.v4i2p103-110

Lions, M., Alvarez, B., & Céspedez, J. (2000). Primer registro de Dipsas indica Laurenti, 1768 (Reptilia, Serpentes, Colubridae) en la Provincia del Chaco, Argentina. *FACENA*, *16*, 121.

Loebmann, D., & de Lema, T. (2012). New data on the distribution of the rare and poorly known Apostolepis goiasensis Prado, 1943 (Serpentes, Xenodontinae, Elapomorphini) with remarks on morphology and colouration. *Herpetology Notes*, *5*(November), 523–525.

López, M. S., & Giraudo, A. R. (2008). Ecology of the snake Philodryas patagoniensis (Serpentes, Colubridae) from northeast Argentina. *Journal of Herpetology*, *42*(3), 474–480. https://doi.org/10.1670/07-087.1

MacCulloch, R. D., Lathrop, A., Kok, P. J. R., Ernst, R., & Kalamandeen, M. (2009). The genus Oxyrhopus (Serpentes: Dipsadidae: Xenodontinae) in Guyana: Morphology, distributions and comments on taxonomy. *Papeis Avulsos de Zoologia*, *49*(36), 487–495. https://doi.org/10.1590/s0031-10492009003600001

Marques, O., Eterovic, A., Nogueira, C., & Sazima, I. (2015). *Serpentes do Cerrado*. Ribeirao Preto: Holos.

Marques, O., Eterovic, A., Strüssmann, C., & Sazima, I. (2005). *Serpentes do Pantanal*. Ribeirao Preto: Holos.

Marques, Otávio Augusto Vuolo, & Puorto, G. (1994). Dieta e comportamento alimentar de Erythrolamprus aesculapii, uma serpente ofiófaga. *Rev. Bras. Biol*, pp. 253–259.

Marques, Otavio A.V., & Muriel, A. P. (2007). Reproductive biology and food habits of the swamp racer Mastigodryas bifossatus from southeastern South America. *Herpetological Journal*, *17*(2), 104–109.

Marques, Otavio A.V., Sawaya, R. J., Stender-Oliveira, F., & Franca, F. G. R. (2006). Ecology of the colubrid snake Pseudablabes agassizii in South-Eastern South America. *Herpetological Journal*, *16*(1), 37–45.

Marques, O. A. V., Almeida-Santos, S. M., Rodrigues, M., & Camargo, R. (2009). Mating and Reproductive Cycle in the Neotropical Colubrid Snake Chironius bicarinatus . *South American Journal of Herpetology*, *4*(1), 76–80. https://doi.org/10.2994/057.004.0110

Marques, O. A. V., Almeida-Santos, S. M., & Rodrigues, M. G. (2006). Activity Patterns in Coral Snakes, Genus Micrurus (Elapidae), in South and Southeastern Brazil. *South American Journal of Herpetology*, *1*(2), 114–120. https://doi.org/10.2994/1808-9798(2006)1[114:apicsg]2.0.co;2

Marques, O. A. V, Eterovic, A., & Sazima, I. (2001). *Serpentes da Mata Atlântica* (p. 184). p. 184.

Martins, M, & Oliveira, M. (1993). The snakes of the genus Atractus Wagler (Reptilia: Squamata: Colubridae) from the Manaus region, central Amazonia, Brazil. *Zoologische Mededelingen*, *67*(1), 21–40.

Martins, Marcio, Marques, O. A. v., & Sazima, I. (2008). How to be arboreal and diurnal and still stay alive: microhabitat use, time of activity, and defense in neotropical forest snakes. *South American Journal of Herpetology*, *3*(1), 58–67. https://doi.org/10.2994/1808-9798(2008)3[58:htbaad]2.0.co;2

Martins, Marcio, & Oliveira, E. (1998). Natural History of snakesin forests of the Manaus Region, Central Amazona, Brazil. *Herpetological Natural History*, *6*(2), 78–150.

Maschio, G. F., da Costa Prudente, A. L., de Lima, A. C., & Feitosa, D. T. (2007). Reproductive Biology of Anilius Scytale (Linnaeus, 1758) (Serpentes, Aniliidae) From Eastern Amazonia, Brazil. *South American Journal of Herpetology*, *2*(3), 179–183. https://doi.org/10.2994/1808-9798(2007)2[179:rboasl]2.0.co;2

Monteiro, C., Montgomery, C. E., Spina, F., Sawaya, R. J., & Martins, M. (2006). Reprodução, alimentação, e Morfologia de Bothrops mattogrossensis (Serpentes, Viperidae, Crotalinae) no Pantanal brasileiro. *Journal of Herpetology*, *40*(3), 408–413. https://doi.org/10.1670/0022-1511(2006)40[408:FRAMOB]2.0.CO;2

Montingelli, G. G., Grazziotin, F. G., Battilana, J., Murphy, R. W., Zhang, Y. P., & Zaher, H. (2019). Higher-level phylogenetic affinities of the Neotropical genus Mastigodryas Amaral, 1934 (Serpentes: Colubridae), species-group definition and description of a new genus for Mastigodryas bifossatus. *Journal of Zoological Systematics and Evolutionary Research*, *57*(2), 205–239. https://doi.org/10.1111/jzs.12262

Myers, C., & Cadle, J. (1994). A New Genus for South American Snakes Related to Rhadinaea obtusa Cope (Colubridae) and Resurrection of Taeniophallus Cope for the “Rhadinaea” brevirostris Group. *Americam Museum Novitates*, *3102*, 1–33. https://doi.org/10.1093/jnci/55.6.1379

Myers, C. W. (1974). The systematics of Rhadinaea (Colubridae), a genus of New World snakes. *Bulletin of the American Museum of Natural History*, *153*, 1.

Nenda, S. (2007). Psomophis genimaculatus (Boettger, 1885) (Serpentes: Colubridae). *Cuadernos de Herpetologia*, *21*(1), 65–66.

Nenda, S. J., & Cacivio, P. M. (2007). Reptilia, Colubridae, Xenodontinae, Lystrophis dorbignyi, Lystrophis pulcher, and Lystrophis semicinctus: distribution extension, new provinces records in Argentina. *Check List*, *3*(2), 126. https://doi.org/10.15560/3.2.126

Nogueira, C., Barbo, F. E., & Ferrarezzi, H. (2012). Redescription of Apostolepis albicollaris Lema, 2002, with a Key for the Species Groups of the Genus Apostolepis (Serpentes: Dipsadidae: Elapomorphini). *South American Journal of Herpetology*, *7*(3), 213–225. https://doi.org/10.2994/057.007.0303

Nogueira, C., Sawaya, R. J., & Martins, M. (2003). Ecology of the Pitviper, Bothrops moojeni, in the Brazilian Cerrado. *Journal of Herpetology*, *37*(4), 653–659. https://doi.org/10.1670/120-02A

Oliveira, B. F., & Scheffers, B. R. (2019). Vertical stratification influences global patterns of biodiversity. *Ecography*, *42*(2), 249. https://doi.org/10.1111/ecog.03636

Parpinelli, L., & Marques, O. A. V. (2008). Seasonal and Daily Activity in the Pale-headed Blindsnake Liotyphlops beui (Serpentes: Anomalepidae) in Southeastern Brazil. *South American Journal of Herpetology*, *3*(3), 207–212. https://doi.org/10.2994/1808-9798-3.3.207

Passos, P, Fernandes, R., Bernils, R. S., & Moura-Leite, J. C. (2010). Taxonomic revision of the Brazilian Atlantic Forest Atractus (Reptilia: Serpentes: Dipsadidae). *Zootaxa*, *2364*(2364), 1–63.

Passos, Paulo, Aguayo, R., & Scrocchi, G. (2009). Rediscovery of the rare Atractus bocki, with assessment of the taxonomic status of Atractus canedii (Serpentes: Colubridae: Dipsadinae). *Journal of Herpetology*, *43*(4), 710–715. https://doi.org/10.1670/08-209.1

Passos, Paulo, & Fernandes, R. (2008). Revision of the Epicrates cenchria complex (Serpentes: Boidae). *Herpetological Monographs*, (22), 1–30. https://doi.org/10.1655/06-003.1

Peters, J. A., & Orejas-Miranda, B. (1972). The Taxonomic Validity of Apostolepis tenuis Ruthven and Apostolepis vittata (Cope) (Serpentes: Colubridae). *Copeia*, *1972*(3), 588. https://doi.org/10.2307/1442938

Pinto, R. R., Passos, P., Portilla, J. R. C., Arredondo, J. C., & Fernandes, R. (2010). Taxonomy of the Threadsnakes of the tribe Epictini (Squamata: Serpentes: Leptotyphlopidae) in Colombia. *Zootaxa*, *28*(2724), 1–28. https://doi.org/10.11646/zootaxa.2724.1.1

Pires, M. G., Da Silva, N. J., Feitosa, D. T., Da Costa Prudente, A. L., Pereira Filho, G. A., & Zaher, H. (2014). A new species of triadal coral snake of the genus Micrurus Wagler, 1824 (Serpentes: Elapidae) from northeastern Brazil. *Zootaxa*, *3811*(4), 569–584. https://doi.org/10.11646/zootaxa.3811.4.8

Pizzatto, L. (2005). Body size, reproductive biology and abundance of the rare pseudoboini snakes genera Clelia and Boiruna (Serpentes, Colubridae) in Brazil. *Phyllomedusa*, *4*(2), 111–122. https://doi.org/10.11606/issn.2316-9079.v4i2p111-122

Pizzatto, L., Almeida-Santos, S. M., & Shine, R. (2007). Life-history adaptations to arboreality in snakes. *Ecology*, *88*(2), 359–366. https://doi.org/10.1890/0012-9658(2007)88[359:LATAIS]2.0.CO;2

Pizzatto, L., Cantor, M., De Oliveira, J. L., Marques, O. A. V., Capovilla, V., & Martins, M. (2008). Reproductive ecology of dipsadine snakes, with emphasis on South American species. *Herpetologica*, *64*(2), 168–179. https://doi.org/10.1655/07-031.1

Pizzatto, L., Jordão, R. S., & Marques, O. A. V. (2008). Overview of reproductive strategies in Xenodontini (Serpentes: Colubridae: Xenodontinae) with new data for Xenodon neuwiedii and Waglerophis merremii. *Journal of Herpetology*, *42*(1), 153–162. https://doi.org/10.1670/06-150R2.1

Pizzatto, L., & Marques, O. A. V. (2006). Interpopulational variation in sexual dimorphism, reproductive output, and parasitism of Liophis miliaris (Colubridae) in the Atlantic forest of Brazil. *Amphibia Reptilia*, *27*(1), 37–46. https://doi.org/10.1163/156853806776052128

Pizzatto, L., & Marques, O. A. V. (2007). Reproductive Ecology of Boine Snakes With Emphasis on Brazilian Species and a Comparison To Pythons. *South American Journal of Herpetology*, *2*(2), 107–122. https://doi.org/10.2994/1808-9798(2007)2[107:reobsw]2.0.co;2

Prieto, Y. A., Giraudo, A. R., & Lpez, M. S. (2012). Diet and sexual dimorphism of Liophis poecilogyrus (Serpentes, Dipsadidae) from the wetland regions of Northeast Argentina. *Journal of Herpetology*, *46*(3), 402–406. https://doi.org/10.1670/10-228

Prudente, A. L., da Silva, F. M., dos Santos Meireles, M., & Puorto, G. (2017). Morphological variation in Siphlophis worontzowi (Squamata: Serpentes: Dipsadidae) from the Brazilian Amazon. *Salamandra*, *53*(2), 245–256.

Puorto, G., & Ferrarezzi, H. (1993). Uma nova especie de Phalotris Cope, 1862, com comentarios sobre o grupo bilineatus (Serpentes: Colubridae: Xenodontinae). *Mem Inst Butantan*, *55*(1), 39–46.

Pyron, R. A., & Burbrink, F. T. (2014). Ecological and evolutionary determinants of species richness and phylogenetic diversity for island snakes. *Global Ecology and Biogeography*, *23*(8), 848–856. https://doi.org/10.1111/geb.12162

Quinteros-Muñoz, O., Peñaranda, D., & Navarro, F. (2010). Rodent consumption by Philodryas psammophidea (Serpentes: Colubridae), from the inter-andean dry valleys of central Bolivia. *Cuadernos de Herpetología*, *24*(2), 129–131. https://doi.org/10.31017/995

Reis Martins, A., Lima Silveira, A., & Freire Bruno, S. (2010). New records of Typhlops brongersmianus (Serpentes, Typhlopidae) in Southeastern Brazil. *Herpetology Notes*, *3*(1), 247–248.

Rosauer, D., Laffan, S. W., Crisp, M. D., Donnellan, S. C., & Cook, L. G. (2009). Phylogenetic endemism: A new approach for identifying geographical concentrations of evolutionary history. *Molecular Ecology*, *18*(19), 4061–4072. https://doi.org/10.1111/j.1365-294X.2009.04311.x

Rossman, D. (1973). Miscellaneous notes on the South American water snake genus Helicops. *Hiss-News-Journal*, *1*(6), 189–191.

Roze, J. (1996). *Coral Snakes of the Americas. Biology, Identification, and Venoms*. Florida: Krieger.

Santos, F. J. M., & Reis, R. E. (2018). Two New Blind Snake Species of the Genus Liotyphlops Peters, 1881 (Serpentes: Anomalepididae), from Central and South Brazil. *Copeia*, *106*(3), 507–514. https://doi.org/10.1643/CH-18-081

Sawaya, R. J., & Sazima, I. (2003). A new species of Tantilla (Serpentes: Colubridae) from southeastern Brazil. *Herpetologica*, *59*(1), 119–126. https://doi.org/10.1655/0018-0831(2003)059[0119:ANSOTS]2.0.CO;2

Scartozzoni, R. R., Trevine, V. C., & Germano, V. J. (2010). Reptilia, Squamata, Serpentes, Dipsadidae, Pseudoeryx plicatilis (Linnaeus, 1758): New records and geographic distribution map. *Check List*, *6*(4), 534–537. https://doi.org/10.15560/6.4.534

Scott, N., Giraudo, A., Scrocchi, G., Aquino, A., Cacciali, P., & Motte, M. (2006). The genera Boiruna and Clelia (Serpentes: Pseudoboini) in Paraguay and Argentina. *Papeis Avulsos de Zoologia*, *46*(2780), 77–105.

Scrocchi, G. (1990a). Contribución al conocimiento de los Leptotyphlopidae de la Argentina II: Nuevos datos sobre Leptotyphlops australis Freibergy Orejas Miranda, 1968. *Acta Zoologica Lilloana*, *39*(2), 113–114.

Scrocchi, G. (1990b). El género Micrurus (Serpentes: Elapidae) en la República Argentina. *Boll. Mus. Reg. Sci. Nat. Torino*, *8*(2), 343–368.

Scrocchi, G., & Cruz, F. (1993). Description of a new species of the genus Lystrophis Cope and a revalidation of Lystrophis pulcher (Jan, 1863) (Serpentes; Colubridae). *Papéis Avulsos de Zoologia*, *38*(10), 171–186.

Scrocchi, G. J., Ferreira, V. L., Giraudo, A. R., Ávila, R. W., & Motte, M. (2005). A new species of Hydrops (Serpentes: Colubridae: Hydropsini) from Argentina, Brazil and Paraguay. *Herpetologica*, *61*(4), 468–477. https://doi.org/10.1655/04-97.1

Scrocchi, G. J., & Giraudo, A. R. (2012). First records of Phalotris sansebastiani Jansen and Kohler, 2008 (Serpentes: Dipsadidae) from Argentina. *Check List*, *8*(5), 900–902. https://doi.org/10.15560/8.5.900

Scrocchi, G., Porto, M., & Rey, L. (1993). Descripción de una especie nueva y situación del género Sibynomorphus (Serpentes: Colubridae) en la Argentina. *Rev. Bras. Biol*, pp. 197–208.

Scrocchi, G., & Viñas, M. (1990). El género Clelia (Serpentes: Colubridae) en la República Argentina: revisión y comentarios. *Boll. Mus. Reg. Sci. Nat. Torino*, *8*(2), 487–499.

Sheehy, C. M., Yánez-Muñoz, M. H., Valencia, J. H., & Smith, E. N. (2014). A new species of Siphlophis (Serpentes: Dipsadidae: Xenodontinae) from the eastern andean slopes of ecuador. *South American Journal of Herpetology*, *9*(1), 30–45. https://doi.org/10.2994/SAJH-D-12-00031.1

Silva, M. V., Souza, M. B., & Bernarde, P. S. (2006). Riqueza e dieta de serpentes no Estado do Acre, Brasil (Reptilia: Serpentes). *Revista Brasileira de Zoociências*, *12*(14), 81.

Silveira, A., & Cotta, G. (2006). Clelia quimi - Distribution. *Herpetological Review*, *37*(2), 242.

Siqueira, D. M., Nascimento, L. P., & Santos-Costa, M. C. dos. (2012). Feeding Biology of Boddaert’s Tropical Racer, Mastigodryas boddaerti (Serpentes, Colubridae) from the Brazilian Amazon. *South American Journal of Herpetology*, *7*(3), 226–232. https://doi.org/10.2994/057.007.0304

Smith, P., Cacciali, P., Scott, N., del Castillo, H., Pheasey, H., & Atkinson, K. (2014). First record of the globally-threatened Cerrado endemic snake Philodryas livida (Amaral, 1923) (Serpentes, Dipsadidae) from Paraguay, and the importance of the Reserva Natural Laguna Blanca to its conservation. *Cuadernos de Herpetología*, *28*(2), 169–171. https://doi.org/10.31017/2277

Strussmann, C., & Sazima, I. (1993). The Snake Assemblage of the Pantanal at Pocone, Western Brazil: Faunal Composition and Ecological Summary. *Studies on Neotropical Fauna and Environment*, Vol. 28, pp. 157–168. https://doi.org/10.1080/01650529309360900

Thomas, R. (1976). *A revision of the South American colubrid snake genus Philodryas Wagler, 1830*. Texas A & M Univ.

Thomas, Robert, & Fernandes, R. (1996). The Systematic Status of Platyinion lividum Amaral, 1923 (Serpentes: Colubridae: Xenodontinae). *Herpetologica*, *52*(2), 271–275.

Tonini, J. F. R., Beard, K. H., Ferreira, R. B., Jetz, W., & Pyron, R. A. (2016). Fully-sampled phylogenies of squamates reveal evolutionary patterns in threat status. *Biological Conservation*, *204*, 23–31. https://doi.org/10.1016/j.biocon.2016.03.039

Torello-Viera, N. F., Araújo, D. P., & Braz, H. B. (2012). Annual and Daily Activity Patterns of the Snail-Eating Snake Dipsas bucephala (Serpentes, Dipsadidae) in Southeastern Brazil. *South American Journal of Herpetology*, *7*(3), 252–258. https://doi.org/10.2994/057.007.0307

Torello-Viera, N. F., & Marques, O. A. V. (2017). Daily activity of neotropical dipsadid snakes. *South American Journal of Herpetology*, *12*(2), 128–135. https://doi.org/10.2994/SAJH-D-16-00023.1

Torres-Carvajal, O., Echevarría, L. Y., Lobos, S. E., Venegas, P. J., & Kok, P. J. R. (2019). Phylogeny, diversity and biogeography of Neotropical sipo snakes (Serpentes: Colubrinae: Chironius). *Molecular Phylogenetics and Evolution*, *130*, 315–329. https://doi.org/10.1016/j.ympev.2018.10.022

Tozetti, A., Oliveira, R., & Pontes, G. (2009). Defensive repertoire of Xenodon dorbignyi (Serpentes, Dipsadidae). *Biota Neotropica*, *9*(3).

Valdujo, P. H., Nogueira, C., & Martins, M. (2002). Ecology of Bothrops neuwiedi pauloensis (Serpentes: Viperidae: Crotalinae) in the Brazilian cerrado. *Journal of Herpetology*, *36*(2), 169–176. https://doi.org/10.1670/0022-1511(2002)036[0169:EOBNPS]2.0.CO;2

Vanzolini, P. E. (1968). Typhlops brongersmai. *Zoologische Mededelingen*, *47*(3), 27–29.

Wilson, L. (1999). Checklist and key to the species of the genus Tantilla with some commentary on distribution. *Smithsonian Herpetological Information Service*, *122*, 3–38.

Yanosky, A. A., & Chani, J. M. (1988). Possible Dual Mimicry of Bothrops and Micrurus by the Colubrid, Lystrophis dorbignyi. *Journal of Herpetology*, *22*(2), 222. https://doi.org/10.2307/1564001

Zaher, H. (1996). A new genus and species of Pseudoboine Snake, with a revision of the genus Clelia (Serpentes, Xenodontinae). *Boll. Mus. Reg. Sci. Nat. Torino*, *14*, 289–337.

Zaher, Hussam, & Caramaschi, U. (1992). Sur le statut taxinomique d’Oxyrhopus trigeminus et O. guibei (Serpentes, Xenodontinae). *Bulletin Du Muséum National d’histoire Naturelle. Section A, Zoologie, Biologie et Écologie Animales*, *14*(3), 805–827.

**Table S2.** Predictor variables used in the study. Climatic variables were download from the WorldClim (Fick & Hijmans, 2017); http:// www.worldclim.org). The annual actual evapotranspiration (AET) was used from (Abatzoglou *et al.*, 2018) and net primary productivity (NPP from https://lpdaac.usgs.gov/, and the Normalized difference vegetation index (NDVI) from (Tucker *et al.*, 2005). Habitat heterogeneity variables were download from http://www.earthenv.org// (Tuanmu & Jetz, 2015), tree cover from the http://earthenginepartners.appspot.com/science-2013-global-forest (Hansen *et al.*, 2013), mean elevation download from https://asterweb.jpl.nasa.gov/gdem.asp (Graham *et al.*, 2014), and soil variables from https://soilgrids.org/ (Hengl *et al.*, 2017).

| **Climatic variables** | |
| --- | --- |
| BIO1 | Annual Mean Temperature |
| BIO2 | Mean Diurnal Range |
| BIO3 | Isothermality (BIO2/BIO7) (* 100) |
| BIO4 | temperature seasonality |
| BIO5 | Max Temperature of Warmest Month |
| BIO6 | Min temperature of coldest month |
| BIO7 | Temperature Annual Range (BIO5-BIO6) |
| BIO8 | Mean Temperature of Wettest Quarter |
| BIO9 | Mean Temperature of Driest Quarter |
| BIO10 | Mean Temperature of Warmest Quarter |
| BIO11 | Mean Temperature of Coldest Quarter |
| BIO12 | Annual Precipitation |
| BIO13 | Precipitation of Wettest Month |
| BIO14 | Precipitation of Driest Month |
| BIO15 | Precipitation Seasonality |
| BIO16 | Precipitation of Wettest Quarter |
| BIO17 | Precipitation of Driest Quarter |
| BIO18 | Precipitation of Warmest Quarter |
| BIO19 | Precipitation of Coldest Quarter |
| **Productivity** | |
| AET | Actual evapotranspiration |
| NPP | Net primary productivity |
| NDVI | Normalized difference Vegetation index |
| **Habitat Heterogeneity** | |
| EVENNESS | Evenness of Enhanced Vegetation Index (EVI) |
| HOMOGENEITY | Similarity of Enhanced Vegetation Index (EVI) |
| TC | Tree cover in the year 2000, canopy closure |
| **Soil** | |
| FRAG | Volumetric fraction of coarse fragments (> 2 mm) |
| SAND | Proportion of sand particles (> 0.05 mm) in the fine earth fraction |
| **Topographic** | |
| ELEV | Mean elevation |

Abatzoglou, J. T., Dobrowski, S. Z., Parks, S. A., & Hegewisch, K. C. (2018). TerraClimate, a high-resolution global dataset of monthly climate and climatic water balance from 1958-2015. *Scientific Data*, *5*, 1–12. https://doi.org/10.1038/sdata.2017.191

Feldman, A., Sabath, N., Pyron, R. A., Mayrose, I., & Meiri, S. (2016). Body sizes and diversification rates of lizards, snakes, amphisbaenians and the tuatara. *Global Ecology and Biogeography*, *25*(2), 187–197. https://doi.org/10.1111/geb.12398

Fick, S., & Hijmans, R. (2017). WorldClim 2: new 1-km spatial resolution climate surfaces for global land areas. *International Journal of Climatology*, 37: 4302–4315. 10.1002/joc.5086

Graham, C. H., Carnaval, A. C., Cadena, C. D., Zamudio, K. R., Roberts, T. E., Parra, J. L., … Sanders, N. J. (2014). The origin and maintenance of montane diversity: Integrating evolutionary and ecological processes. *Ecography*, *37*(8), 711–719. https://doi.org/10.1111/ecog.00578

Hansen, M. C., Potapov, P. v., Moore, R., Hancher, M., Turubanova, S. A., Tyukavina, A., … Townshend, J. R. G. (2013). High-resolution global maps of 21st-century forest cover change. *Science*, *342*(6160), 850–853. https://doi.org/10.1126/science.1244693

Hengl, T., de Jesus, J. M., Heuvelink, G. B. M., Gonzalez, M. R., Kilibarda, M., Blagotić, A., … Kempen, B. (2017). SoilGrids250m: Global gridded soil information based on machine learning. In *PLoS ONE* (Vol. 12). https://doi.org/10.1371/journal.pone.0169748

Oliveira, B. F., & Scheffers, B. R. (2019). Vertical stratification influences global patterns of biodiversity. *Ecography*, *42*(2), 249. https://doi.org/10.1111/ecog.03636

Tuanmu, M. N., & Jetz, W. (2015). A global, remote sensing-based characterization of terrestrial habitat heterogeneity for biodiversity and ecosystem modelling. *Global Ecology and Biogeography*, *24*(11), 1329–1339. https://doi.org/10.1111/geb.12365

Tucker, C. J., Pinzon, J. E., Brown, M. E., Slayback, D. A., Pak, E. W., Mahoney, R., … el Saleous, N. (2005). An extended AVHRR 8-km NDVI dataset compatible with MODIS and SPOT vegetation NDVI data. *International Journal of Remote Sensing*, *26*(20), 4485–4498. https://doi.org/10.1080/01431160500168686

**Table S3.** variance inflation factor (VIF) and Pearson correlation test of the variables using in the analysis.

|  | **Environmental variables** | **VIF** |
| --- | --- | --- |
| Climatic | Annual mean temperature | 5 |
|  | Precipitation seasonality | 2 |
| Habitat Heterogeneity | Evenness of the Enhaced Vegetation Index (EVI) | 2 |
|  | Homogeneity of the Enhaced Vegetation Index (EVI) | 3 |
|  | Tree cover | 2 |
| Productivity | Actual evapotranspiration | 2 |
|  | Net primary productivity | 2 |
|  | Normalized difference vegetation index | 2 |
| Soil | Volumetric percentage of coarse fragments | 3 |
|  | Proportion of sand particles in the soil | 1 |

**Table S4.** Pearson correlation test for all the 10 environmental variables. No correlation between variables cor >0.8.

|  | Annual mean temperature | Precipitation seasonality | Evenness of the Enhaced Vegetation Index (EVI) | Actual evapotranspiration | Volumetric percentage of coarse fragments | Homogeneity of the Enhaced Vegetation Index (EVI) | Net primary productivity | Proportion of sand particles in the soil | Tree cover | Normalized difference vegetation index |
| --- | --- | --- | --- | --- | --- | --- | --- | --- | --- | --- |
| Annual mean temperature | 1.0 | 0.0 | 0.5 | 0.1 | -0.5 | 0.5 | -0.1 | 0.0 | 0.6 | 0.5 |
| Precipitation seasonality | 0.0 | 1.0 | 0.2 | -0.3 | 0.5 | 0.5 | -0.2 | 0.2 | 0.1 | -0.3 |
| Evenness of the Enhaced Vegetation Index (EVI) | 0.5 | 0.2 | 1.0 | 0.5 | -0.2 | 0.1 | 0.2 | -0.2 | 0.1 | 0.2 |
| Actual evapotranspiration | 0.1 | -0.3 | 0.5 | 1.0 | -0.3 | -0.4 | 0.4 | -0.4 | 0.0 | 0.3 |
| Volumetric percentage of coarse fragments | -0.5 | 0.5 | -0.2 | -0.3 | 1.0 | 0.1 | 0.2 | 0.2 | -0.4 | -0.4 |
| Homogeneity of the Enhaced Vegetation Index (EVI) | 0.5 | 0.5 | 0.1 | -0.4 | 0.1 | 1.0 | -0.4 | 0.3 | 0.3 | 0.0 |
| Net primary productivity | -0.1 | -0.2 | 0.2 | 0.4 | 0.2 | -0.4 | 1.0 | -0.3 | -0.2 | 0.0 |
| Proportion of sand particles in the soil | 0.0 | 0.2 | -0.2 | -0.4 | 0.2 | 0.3 | -0.3 | 1.0 | 0.2 | 0.1 |
| Tree cover | 0.6 | 0.1 | 0.1 | 0.0 | -0.4 | 0.3 | -0.2 | 0.2 | 1.0 | 0.5 |
| Normalized difference vegetation index | 0.5 | -0.3 | 0.2 | 0.3 | -0.4 | 0.0 | 0.0 | 0.1 | 0.5 | 1.0 |

**Figure S1.** Principal components of our trait data. Fossorial species are located at the left and arboreal species at right.


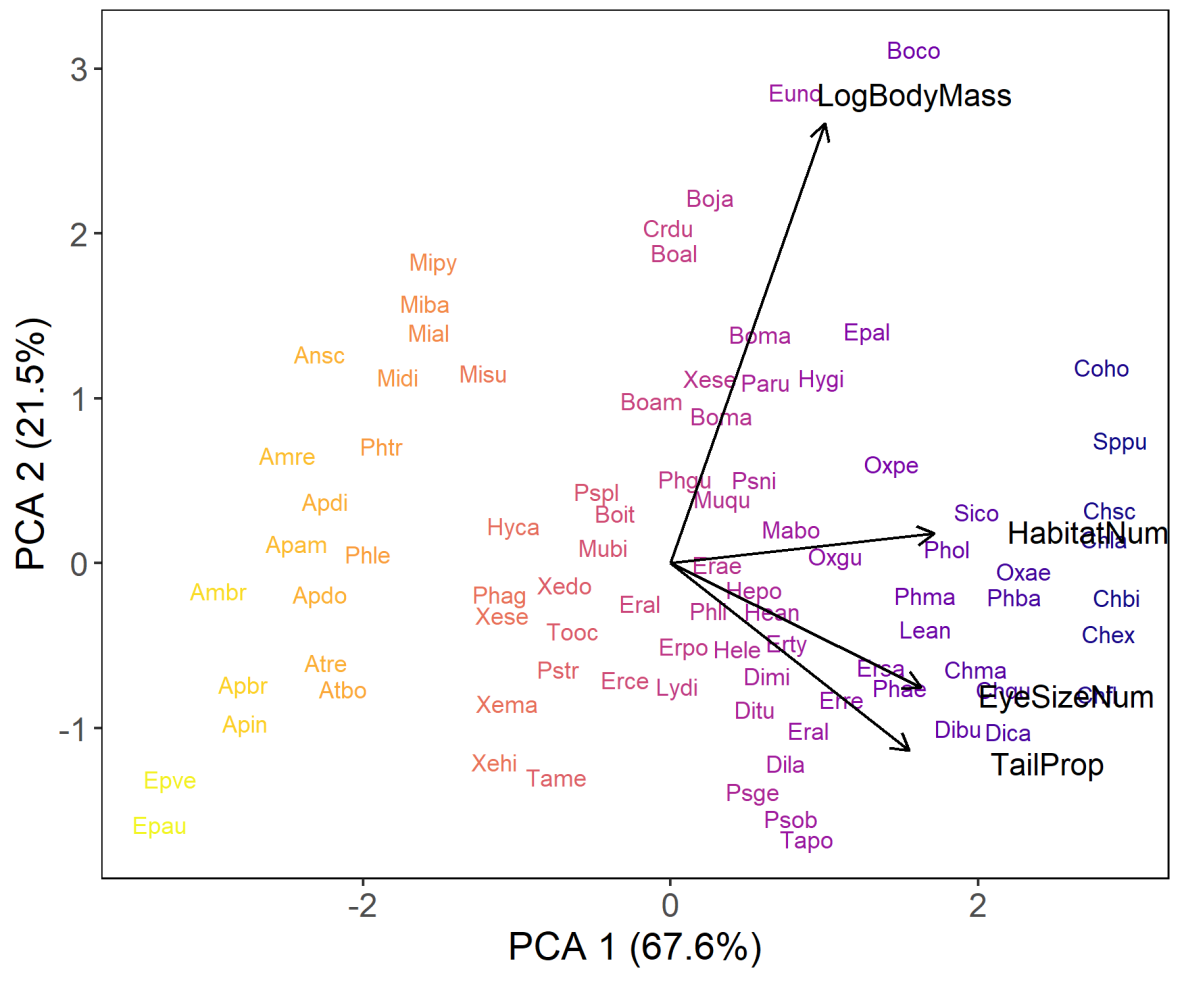


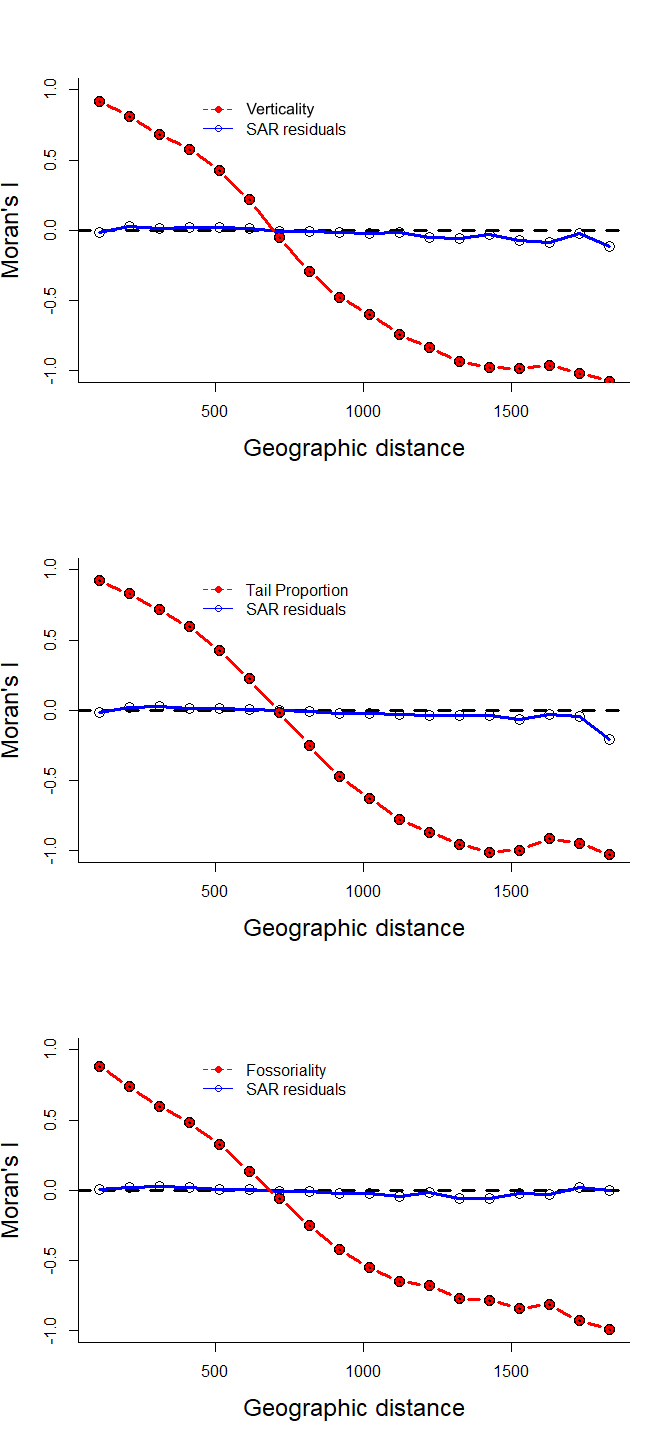
**Figure S2.** Regional autocorrelograms. Red line represents spatial autocorrelation in vertical habitat, tail proportion and fossoriality. of our response and predictors variables. Blue lines are the spatial autocorrelation in our spatial autoregressive (SAR) models. Averaged residuals represent the difference between raw values from an explanatory variable and the averaged fitted values (weighted AICc x fitted values) from SAR models.


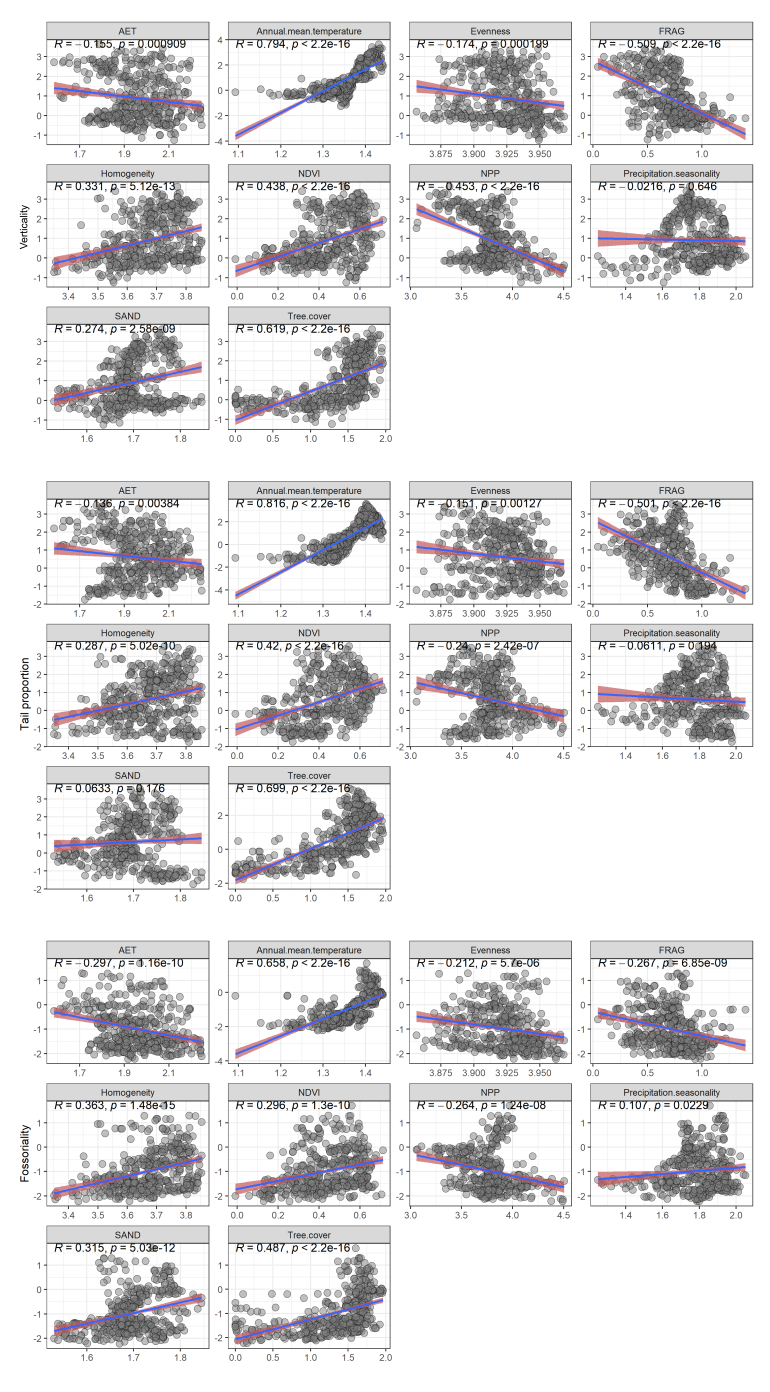
**Figure S3.** Correlation between our response and predictors variables. Numbers on top represent Pearson correlation with significance probability.


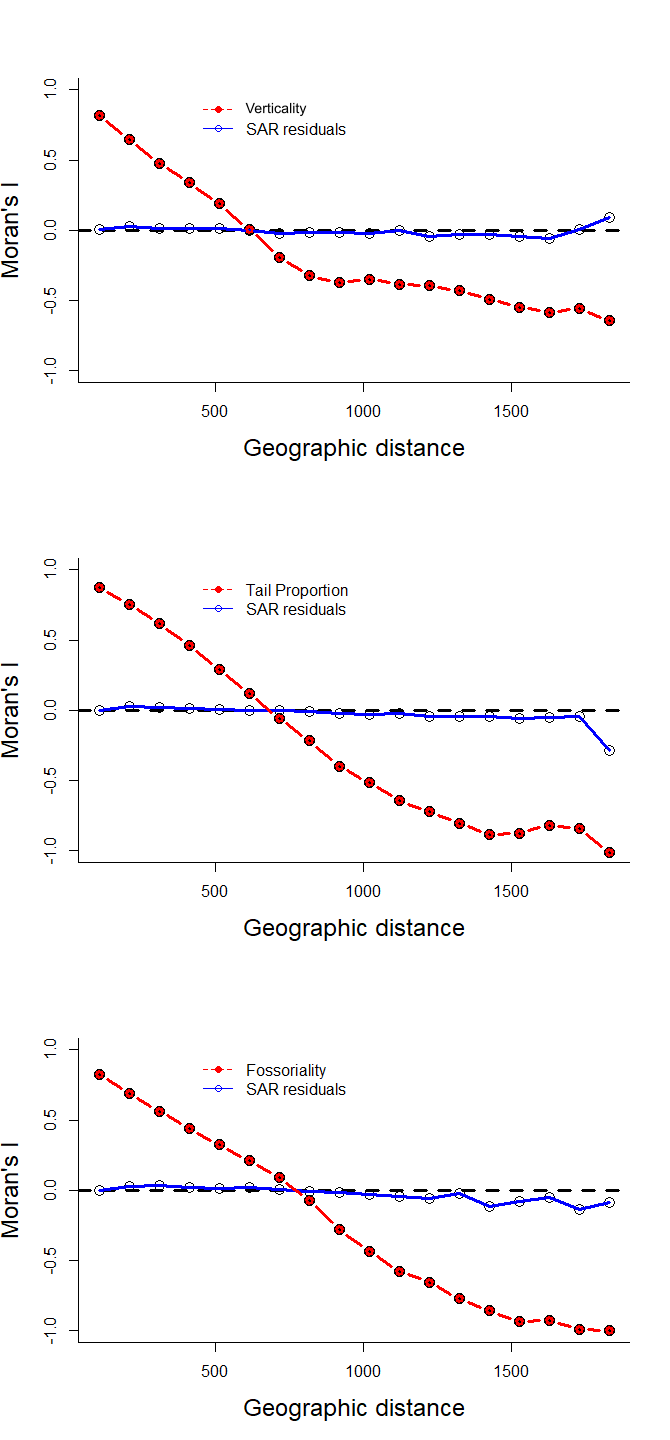
**Figure S4.** Regional autocorrelograms from our sensitivity analysis. Red line represents spatial autocorrelation in vertical habitat, tail proportion and fossoriality. of our response and predictors variables. Blue lines are the spatial autocorrelation in our spatial autoregressive (SAR) models. Averaged residuals represent the difference between raw values from an explanatory variable and the averaged fitted values (weighted AICc x fitted values) from SAR models.


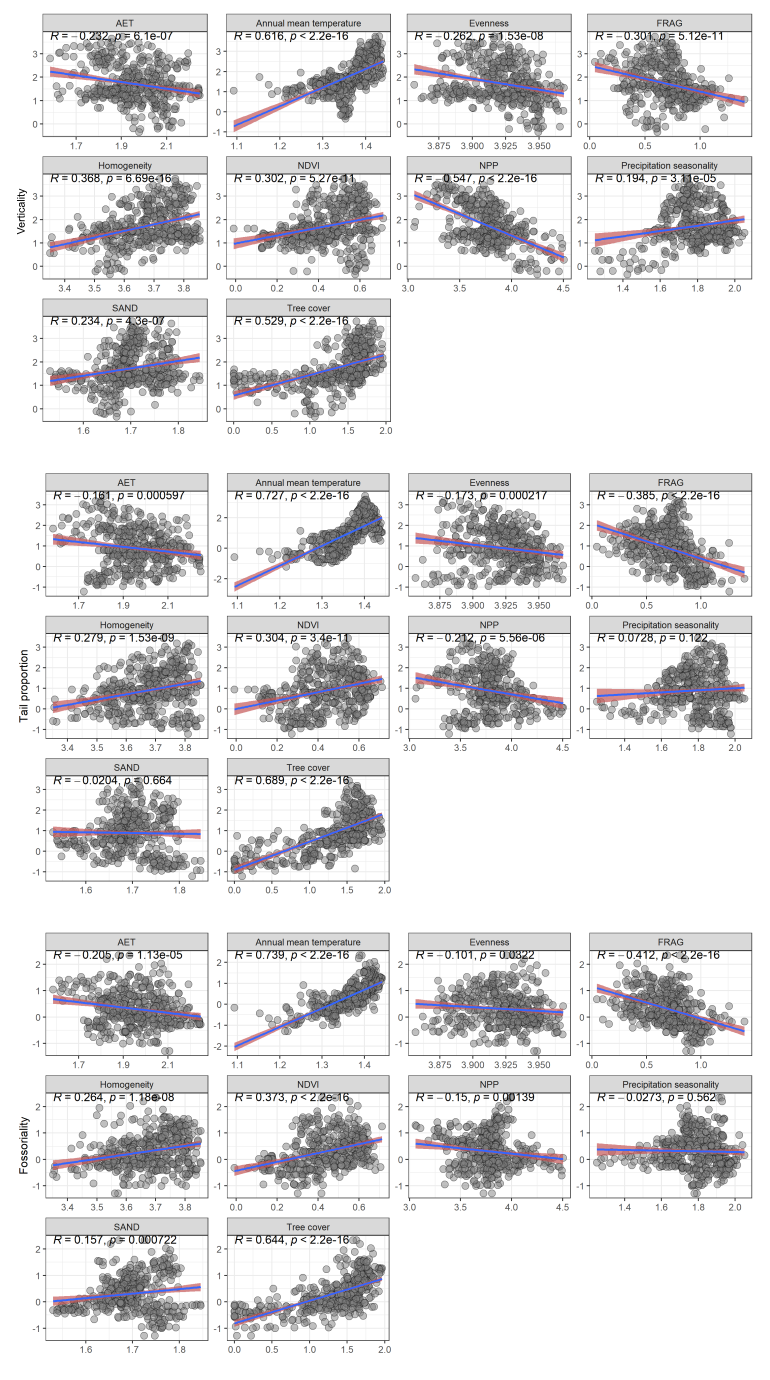
**Figure S5.** Correlation between our response and predictors variables from our sensitivity analysis. Numbers on top represent Pearson correlation with significance probability.

**Figure S6.** The threshold of explained variances to identify the optimal number of phylogenetic regions. Red line and circle in the graph represent the optimal numbers of phylogenetic region.


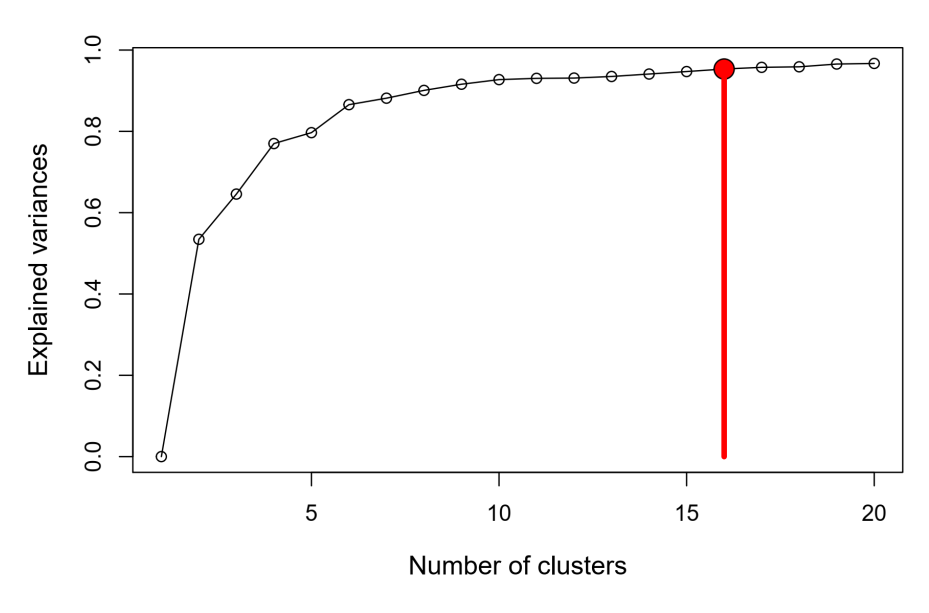


**Figure S7.** Geographical pattern of phylogenetic endemism PE, phylogenetic diversity PD, phylogenetic species variability (PSV). Phylogenetic endemism measures endemism based on the relatedness of species (Rosauer *et al.*, 2009; Daru *et al.*, 2020), calculate the spatial uniqueness of each branch in the tree taking the reciprocal of its range, multiplying by branch length, and summing for all branch lengths present at a sample/site (Rosauer *et al.*, 2009). Phylogenetic diversity is a measure to compared diversity in geographic areas, evolutionary history shared between areas (Graham & Fine, 2008; Laffan *et al.*, 2016), and describe the evolutionary distinctiveness of component taxa (Faith, 1992; Helmus *et al.*, 2007). How phylogenetically related are species in a community was measure through phylogenetic species variability (Helmus *et al.*, 2007). PSV is standardized to vary between zero when species are closely related and one when species are distantly related, indicating maximum variability. As relatedness increases, the index approaches 0, indicating reduced variability (Helmus *et al.*, 2007; Pyron & Burbrink, 2014; Burbrink & Myers, 2015).


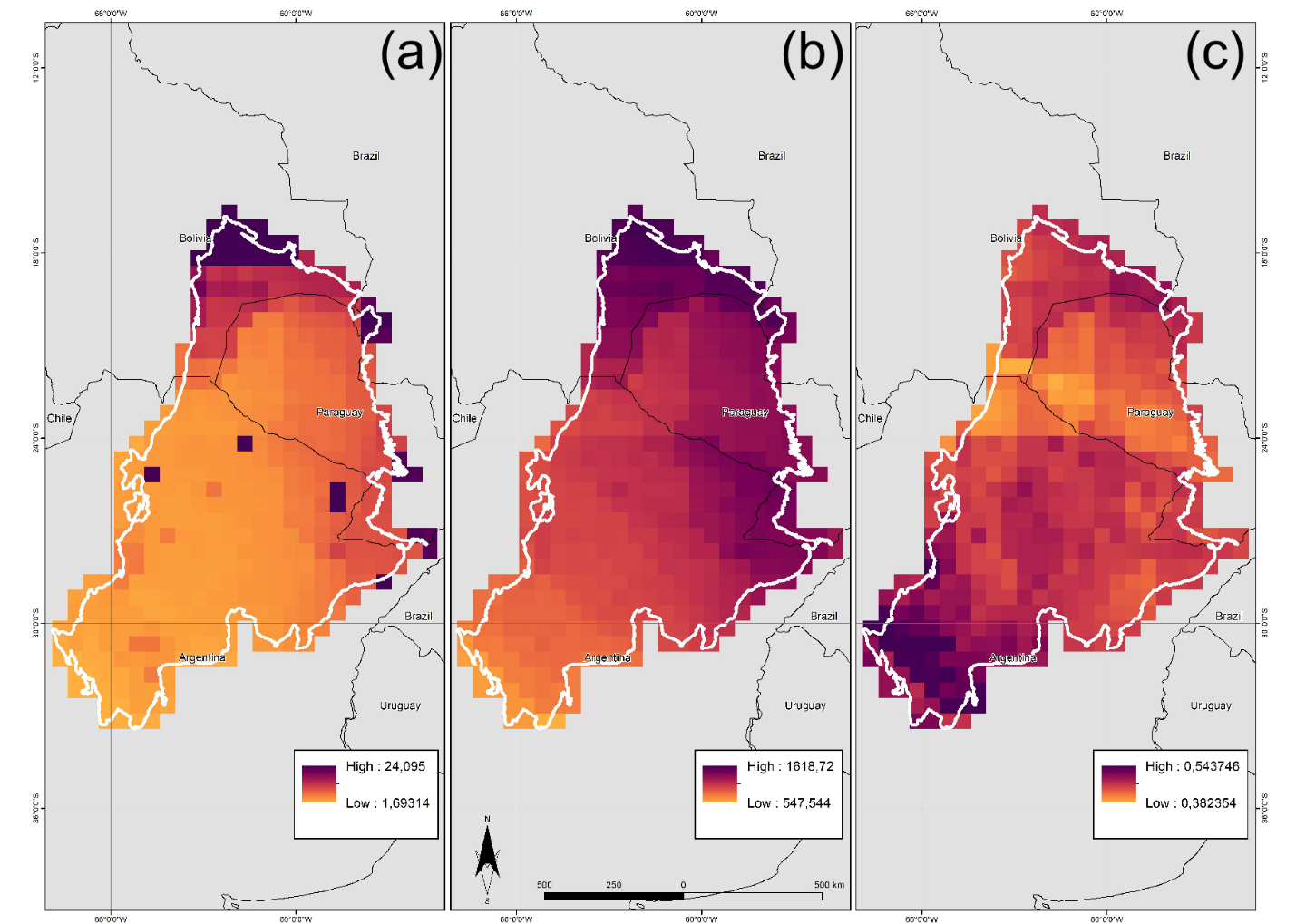


Burbrink, F. T., & Myers, E. A. (2015). Both traits and phylogenetic history influence community structure in snakes over steep environmental gradients. *Ecography*, *38*(10), 1036–1048. https://doi.org/10.1111/ecog.01148

Daru, B. H., Karunarathne, P., & Schliep, K. (2020). phyloregion: R package for biogeographical regionalization and macroecology. *Methods in Ecology and Evolution*, *11*(11), 1483–1491. https://doi.org/10.1111/2041-210X.13478

Faith, D. (1992). Conservation evaluation and phylogenetic diversity. *Biological Conservation*, *61*, 1–10. https://doi.org/10.1016/0003-2697(75)90168-2

Graham, C. H., & Fine, P. V. A. (2008). Phylogenetic beta diversity: Linking ecological and evolutionary processes across space in time. *Ecology Letters*, *11*(12), 1265–1277. https://doi.org/10.1111/j.1461-0248.2008.01256.x

Helmus, M. R., Bland, T. J., Williams, C. K., & Ives, A. R. (2007). Phylogenetic measures of biodiversity. *The American Naturalist*, *169*(3). https://doi.org/10.1086/511334

Laffan, S. W., Rosauer, D. F., di Virgilio, G., Miller, J. T., González-Orozco, C. E., Knerr, N., … Mishler, B. D. (2016). Range-weighted metrics of species and phylogenetic turnover can better resolve biogeographic transition zones. *Methods in Ecology and Evolution*, *7*(5), 580–588. https://doi.org/10.1111/2041-210X.12513

Pyron, R. A., & Burbrink, F. T. (2014). Early origin of viviparity and multiple reversions to oviparity in squamate reptiles. *Ecology Letters*, *17*(1), 13–21. https://doi.org/10.1111/ele.12168

Rosauer, D., Laffan, S. W., Crisp, M. D., Donnellan, S. C., & Cook, L. G. (2009). Phylogenetic endemism: A new approach for identifying geographical concentrations of evolutionary history. *Molecular Ecology*, *18*(19), 4061–4072. https://doi.org/10.1111/j.1365-294X.2009.04311.x

Figure S8. Phylogeny of snakes from the Gran Chaco, representing the 140 species used in this study. Phylogeny data was extracted and pruned from Tonini et al. (2016).


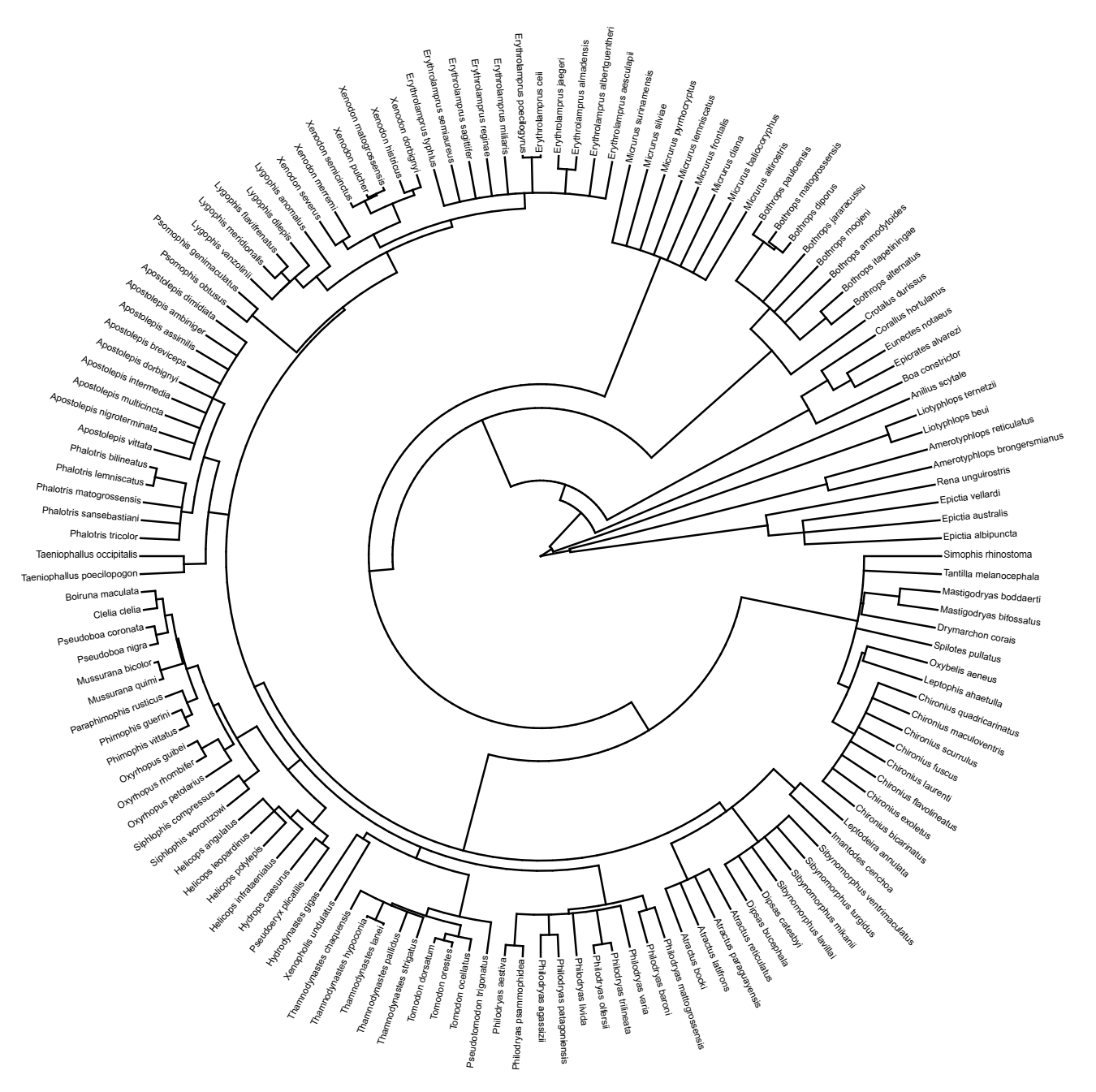


Tonini, J. F. R., Beard, K. H., Ferreira, R. B., Jetz, W., & Pyron, R. A. (2016). Fully-sampled phylogenies of squamates reveal evolutionary patterns in threat status. *Biological Conservation*, *204*, 23–31. https://doi.org/10.1016/j.biocon.2016.03.039
